# Plying potency assays for immunotherapy of solid tumors

**DOI:** 10.3389/fimmu.2026.1770222

**Published:** 2026-03-24

**Authors:** Jorge S. Burns, Riana Gjeta, Petr Lesný, Joaquim Vives

**Affiliations:** 1 Department of Environmental and Prevention Sciences, University of Ferrara, Ferrara, Italy; 2 National Blood Transfusion Center, Tirana, Albania; 3 Department of Immunotherapy, Institute of Hematology and Blood Transfusion, Prague, Czechia; 4 Banc de Sang i Teixits, Barcelona, Spain; 5 Vall d’Hebron Institut de Recerca, Barcelona, Spain; 6 Departament de Medicina, Universitat Autònoma de Barcelona, Cerdanyola del Vallès, Barcelona, Spain

**Keywords:** CAR T-cell therapy, axicabtagene ciloleucel, CAR-engineered immune cell platforms, 3D tumor culture models, spatial biology, artificial intelligence, regulatory frameworks for advanced therapies, potency assays

## Abstract

Potency assays for cellular immunotherapies have advanced considerably yet remain only partially aligned with the complex requirements of solid tumors, where trafficking, persistence, metabolic fitness and spatially constrained effector function are key determinants of *in vivo* performance. Critical quality attributes and mechanism of action can be used to anchor more informative potency strategies for tumor-infiltrating lymphocytes and CAR-engineered platforms, including CAR T, CAR-NK, CAR-M and CAR-γδ T cells. Emerging three-dimensional models, spatial biology, label-free real-time technologies and AI-enabled analytics are examined as routes to integrate microenvironmental stressors and dynamic single-cell behavior into assay design. A “plying” framework is proposed to organize potency assessment into layered, iteratively refined panels that span lean, regulatory compliant release testing through to comprehensive exploratory profiling, providing a practical path toward clinically relevant and regulatorily acceptable potency assurance for solid tumor immunotherapy products.

## Introduction

**Figure 1:**
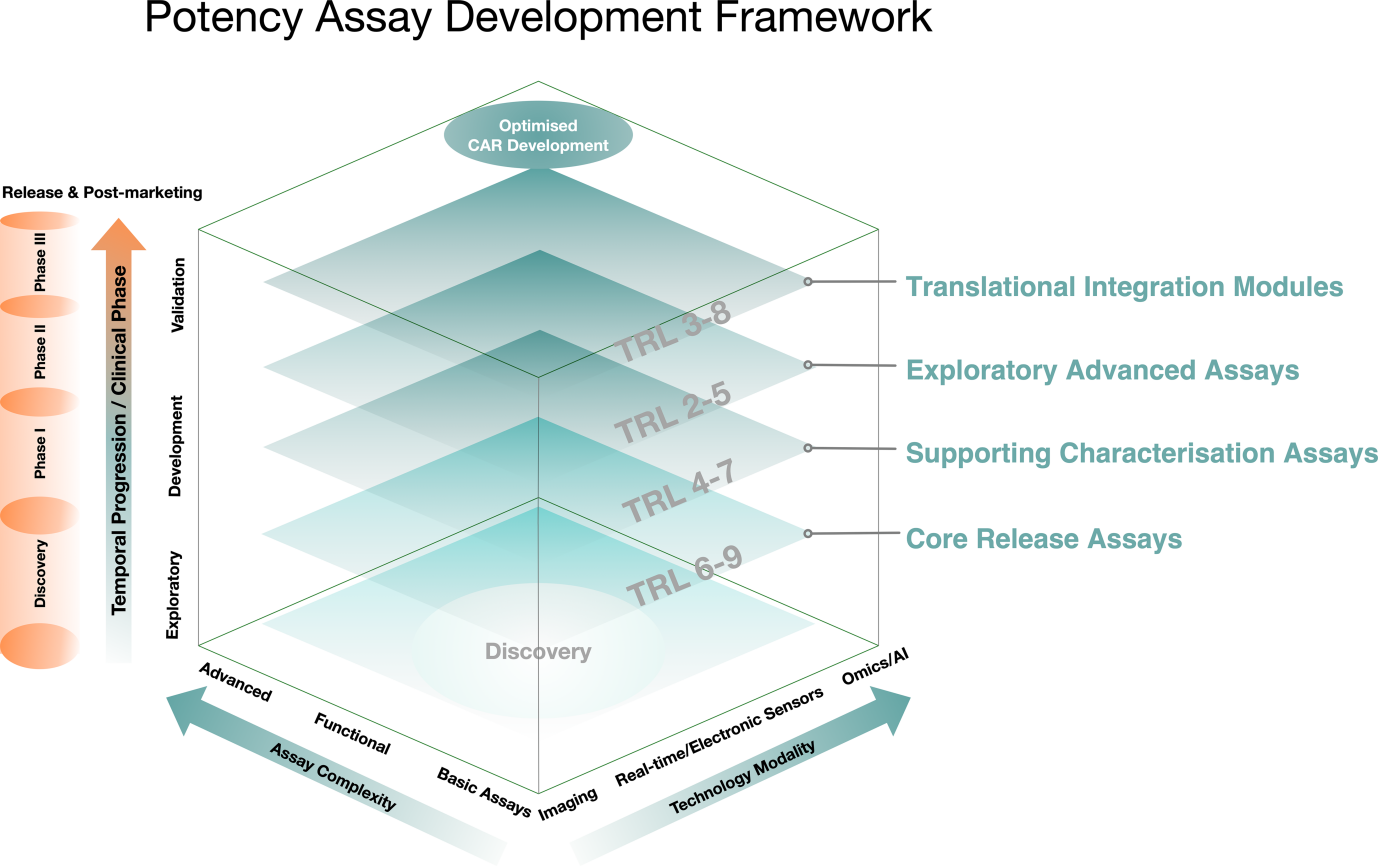
Plying framework for phased potency assay evolution. Conceptual cube illustrating how potency assays are layered over the course of cellular immunotherapy development, from a Discovery base through clinical validation and lifecycle use. The vertical axis (Temporal Progression) aligns assay deployment with development stage and clinical phase, from preclinical exploration and Phase I feasibility to Phase II/III confirmation and post−approval refinement. Above the Discovery base, four tiers are depicted: Foundational Core Release Assays providing CQA−anchored, MOA−linked release criteria; Supporting Characterisation Assays capturing identity, phenotypic and basic functional attributes; Exploratory Advanced Assays incorporating higher−order technologies such as 3D models, spatial profiling, label−free real−time platforms and AI−enabled analytics; and a Translational Integration Modules layer that uses multi−modal models, digital manufacturing intelligence and real−world evidence to connect *in vitro* measurements with *in vivo* outcomes. Phase bands indicate typical periods of predominant use and promotion between tiers; tiers are not synonymous with clinical phases. The horizontal axis (Assay Complexity) spans single−parameter tests to multi−parametric, systems−level readouts, while the depth axis (Technology Modality) extends from conventional methods to New Approach Methodologies (NAMs) and integrated digital twins. Together, these dimensions require explicit categorization of each assay into a tier, link promotion between tiers to rules of evidence (e.g. demonstrated MOA−consistent clinical correlations), and tie assay evolution to phase−appropriate regulatory expectations for potency assurance. “Optimized development” is achieved when temporal readiness, technological sophistication and analytical depth are aligned at each phase to deliver clinically relevant, regulatorily defensible potency assessment.

The term “plying” encompasses multifaceted meanings that collectively illuminate modern immunotherapy development. At its core, plying represents the consistent, methodical use of tools, directly paralleling the systematic application of analytical methodologies in cellular immunotherapy characterization (1). Plying also embodies the persistent questioning and ongoing revision that drives scientific discovery, relevant to probing the complex mechanisms underlying immune T-cell activation, expansion, and therapeutic efficacy (2). Plying as insistent feeding, mirrors ex vivo T-cell manufacturing’s critical feeding strategies, where precise nutrient delivery and cytokine supplementation determine therapeutic cell quality and potency (3). A ply, a single layer within a composite, evokes the multi-layered architecture of solid tumors and their microenvironments, where myriad physical barriers, immunosuppressive networks and cellular heterogeneity together create formidable therapeutic challenges (4). The game theory interpretation of ‘ply’ as the count of individual layers within branching decision trees alludes to strategic frameworks for navigating immunotherapy development, from target selection to clinical implementation (5, 6). This plying paradigm demands sophisticated analytical approaches systematically capturing the multidimensional aspects of cellular immunotherapies. For example, three-dimensional (3D) solid tumors require more than cell monolayer assays to recapitulate the spatial complexity and temporal dynamics that may define therapeutic success (7). Multi-layered analytical platforms extend potency measurement beyond simple endpoints, enabling comprehensive real-time assessment within more physiologically relevant contexts (8); adopting the plying principle through systematic, iterative refinement across multiple analytical layers. The risk-based strategy guiding ATMP development (9), reflects this ‘plying’ philosophy, granting manufacturers a degree of methodological flexibility in early clinical phases where knowledge is sparse and iterative learning dominates. However, as development progresses from Phase I toward pivotal trials, manufacturers are expected to progressively strengthen their testing strategy, incorporating functional assays to provide adequate quality control. Such progression necessitates that potency assays evolve from basic proof-of-concept tests into full-fledged, multi-parametric evaluations that are closely aligned with the product’s mechanism of action (MOA) and capable of supporting an informed assessment of clinical relevance. Operationally, we interpret “plying” as the structured layering of potency assays according to their regulatory purpose, information depth and readiness for clinical decision−making. This layered scheme maps broadly onto clinical development phases and technology readiness level (TRL) bands adapted for health-intervention maturity assessment (10) (**Figure 1**). In practice, the plying framework organizes assays into four tiers that evolve with product development: (i) a Foundational Core tier comprising validated, mechanism of action (MOA)−linked potency assays used for lot release and comparability; (ii) Supporting Characterisation assays that deepen understanding of identity, phenotype and basic function; (iii) Exploratory Advanced assays incorporating higher−order technologies including New Approach Methodologies (NAMs) such as 3D models, spatial profiling, label−free real−time platforms, multi−omic signatures and AI—enabled analytics to generate mechanistic and predictive insights; and (iv) a Translational Integration layer that connects *in vitro* measurements with *in vivo* pharmacodynamic readouts, digital manufacturing intelligence and real−world evidence. For example, in a typical CD19 CAR T−cell program, a flow−cytometric or real−time impedance−based cytotoxicity assay quantifying target−cell killing across effector−to−target ratios would be established and validated as a MOA−linked core potency assay in the Foundational Core tier and used for release and comparability throughout development and post−approval. In parallel, integrated multi−omic profiling of the CAR T−cell product and patient samples (e.g. transcriptomic, proteomic and immune−phenotypic signatures associated with expansion, persistence and toxicity) would initially sit in the Exploratory Advanced tier to discover candidate biomarkers, with selected signatures subsequently promoted into the Translational Integration layer once they show reproducible correlation with clinical outcomes and are embedded in standardized analysis pipelines. In this manner, plying frameworks for individual CAR T−cell programs provide a stepwise, risk−based path for assay maturation, ensuring that increasingly complex, multi−parametric readouts are only advanced to higher tiers when supported by evidence of technical robustness, clinical relevance and regulatory fitness−for−purpose. Hence, regulatory expectations mirror the conceptual architecture of plying, with early flexibility maturing stepwise into a stratified, rigorous and interlocked quality−control structure.

## Literature synthesis and scope

This narrative review synthesizes peer-reviewed literature on potency assays for solid tumor immunotherapies (published through December 2025), regulatory guidance from FDA, EMA, and ICH, and clinical trial data for approved products (lifileucel, tisagenlecleucel, axicabtagene ciloleucel). Literature identification employed comprehensive database searches (PubMed/MEDLINE, Web of Science, Scopus) and generative AI assistance (Perplexity AI) to identify emerging technologies. Primary search terms included (“potency assay” OR “functional assay”) AND (“CAR-T” OR “TIL” OR “CAR-NK”) AND (“solid tumor/tumour”), supplemented by targeted searches for emerging technologies (3D culture, organoids, spatial biology, label-free monitoring, artificial intelligence). All content was critically reviewed and verified by the author. Given the heterogeneous nature of potency assay technologies and absence of standardized outcome measures, narrative synthesis was employed rather than meta-analysis. Literature is organized thematically by cell therapy platform (TIL, CAR T, CAR-NK, CAR-M, CAR-γδ T cells), assay technology (functional, phenotypic, 3D culture, spatial biology, label-free, AI-enabled), and development stage (exploratory, qualified, validated), with emphasis on validation rigor, clinical correlation, and regulatory acceptance. Studies were reviewed with emphasis on 2020–2025 publications; seminal earlier studies were retained for historical context and mechanistic understanding.

## Broadening immunotherapy to solid tumours

Cellular immunotherapy aims to advance beyond unprecedented hematological malignancy successes achieved with tumor-infiltrating lymphocytes (TIL) and chimeric antigen receptor (CAR) T-cells into the realm of solid tumor treatment (11). Solid malignancies present unique potency assessment challenges: physical architecture creates T-cell infiltration barriers, while immunosuppressive microenvironments counteract therapeutic function through checkpoint inhibition, metabolic competition, and regulatory cell recruitment (12).

Melanoma has emerged as the vanguard solid tumor model, with inherent immunogenicity, well-characterized tumor-associated antigens, and established clinical protocols advancing immunotherapy development (13, 14). Clinical data from melanoma TIL therapy trials provided evidence for potency-outcome correlations. In the C-144–01 study of lifileucel (Amtagvi), the first FDA-approved TIL therapy and first cellular therapy for any solid tumor, patients achieved objective responses: 80% showed disease control and 36% achieved tumor shrinkage including two complete responses (15) found to be durable (13). Lessons learned from melanoma immunotherapy are being extended to pancreatic adenocarcinoma, glioblastoma, and treatment-resistant solid tumors (16, 17).

Current clinical outcomes, while promising, remain inconsistent across populations and tumor types, underscoring critical need for biomarkers predicting solid tumor microenvironment performance (18–20). The translation of preclinical biomarkers into clinically relevant potency assays remains a fundamental challenge for cellular immunotherapy. The complexity is exemplified by the programmed cell death protein 1 (PD-1) test case in tumor-infiltrating lymphocyte (TIL) therapy. Seminal studies demonstrated that CD8+PD-1+ lymphocytes from fresh tumor digests were enriched for tumor-reactive T-cell (21, 22) and subsequent correlative analysis supported that high PD-1+ TIL infiltration was associated with improved responses to checkpoint inhibitors in multiple tumor types (23). Such observations provided compelling mechanistic rationale for incorporating PD-1 selection into TIL manufacturing. However, direct comparison of selected versus unselected T-cells did not show the anticipated superiority; CD8+-enriched TIL yielded no better response rates compared with bulk-expanded products in melanoma (24) and PD-1 selected TIL did not demonstrate clear efficacy advantages in head and neck cancer, albeit in a limited patient cohort (25). Meanwhile, lifileucel, a bulk-expanded, unselected preparation, achieved durable responses in about one-third of the melanoma patients. This apparent paradox, where a biomarker predicts response yet selecting for that biomarker does not enhance efficacy, highlights involvement of multiple factors beyond tumor reactivity, including persistence, polyclonality, helper cell support, metabolic fitness and resistance to the immunosuppressive microenvironment. Immunotherapy potency reconceptualization for solid tumors requires evaluation beyond direct cytotoxic contact; evaluation must also include trafficking capability, barrier penetration, persistence within hostile microenvironments and sustained functionality despite immunosuppressive pressures (19, 26). Sophisticated potency assessment frameworks that define how to measure these multifactorial determinants of *in vivo* behavior, integrating functional and phenotypic assessment, are the subject of this review (**Figure 1**).

## Critical quality attributes, mechanism of action and efficacy

Regulatory frameworks emphasize the fundamental association between an understanding of the MOA and potency assurance. Critical Quality Attributes (CQAs) represent measurable characteristics controlling product quality, safety, and efficacy (27). For cellular immunotherapies, these encompass identity, purity, potency, and safety parameters (28). Regulatory guidance recognizes that potency measurements must reflect biological activity relevant to the intended therapeutic effects (29). For TIL therapies, the MOA encompasses tumor recognition, activation, proliferation, and sustained cytotoxic function within the microenvironment, requiring multiple analytical platforms (30). CAR T-cell therapies present distinct MOA considerations, with engineered receptor specificity, intracellular signaling dynamics, and persistence characteristics defining CQA (31, 32). Critical quality attributes (CQAs) should ideally be assessed on the final product; however, the unique characteristics of ATMP manufacturing may necessitate evaluating certain CQAs in key intermediates or, for cryopreserved cell products, in post-thaw samples that recapitulate the final-use condition. In all cases, the manufacturer must demonstrate and validate that the chosen potency assays are relevant and appropriately reflect the product’s intended biological activity (9).

The MOA-potency-clinical efficacy relationship represents a fundamental development challenge. Complexity arises from the multifactorial nature of what is needed for therapeutic success, where product potency, patient factors, and tumor characteristics interact (33). Multi-omics approaches demonstrate that single-parameter potency measurements fail to capture the multidimensional therapeutic aspects needed for success; composite potency signatures incorporating functional, phenotypic, and molecular characteristics provide more robust clinical predictors (34, 35). Temporal dimensions are significant: for example, Day-5 products may retain stem-like, metabolically active phenotypes whereas day-10 products may show terminal differentiation, demonstrating cell expansion-limited functional fitness (36). Kinetic potency measurements and predictive modeling improve extrapolation from *in vitro* to *in vivo* performance (37, 38). Patient-specific factors suggest universal potency thresholds may be insufficient (39), with AI/machine learning integration offering unprecedented opportunities for strengthening connections between ex vivo metrics and *in vivo* consequences (40).

The translation of CQAs into quantitative, validated acceptance criteria has been exemplified by FDA−approved cellular therapies such as tisagenlecleucel (Kymriah) and lifileucel (Amtagvi). For Kymriah, lot−release specifications operationalized multiple CQAs: identity and purity were defined by CAR expression and T−cell subset composition by flow cytometry; potency was assessed by IFN−γ release upon co−culture with CD19^+^ target cells, with numerical acceptance criteria derived from phase 2/3 clinical material; safety was ensured by limits on vector copy number, absence of replication−competent lentivirus, and compendial sterility/endotoxin tests; while viability was controlled by minimum viable−cell thresholds (41). Lifileucel, the first approved TIL therapy for melanoma, similarly coupled identity (CD3^+^ T−cell content), purity (absence of tumor cells) and viability thresholds with MOA−aligned potency readouts based on tumor−reactive cytokine production (IFN−γ and/or TNF−α, 4−1BB up−regulation) and a defined viable TIL dose range. Long−term follow−up from the Phase 2 C−144−01 trial (IRC−assessed objective response rate (ORR) 31.4%, median duration of response (DOR) not reached at a median 27.6−month follow−up, with 41.7% of responses maintained for ≥18 months) supported the adequacy of this potency assurance strategy for an unselected bulk TIL product (13). Together, these products illustrated how MOA−relevant functional assays, combined with orthogonal identity, purity, viability and safety attributes, could be statistically anchored to clinical trial material to define CQA−based acceptance criteria that subsequently underpinned routine lot−release specifications.

## TIL and CAR T-cell based immunotherapy potency assessment

TIL are naturally occurring T cells within solid malignancies, embodying endogenous immune responses (42). Pre-selected, tumor-experienced lymphocytes can be isolated, expanded ex vivo, and reinfused in large numbers sufficient to overcome immunosuppressive microenvironments. CD8+ enrichment for tumor-specific clonotypes (43) provides broad tumor reactivity (44), although clinical advantages of selection strategies are still being defined. Therapeutic product manufacture involves successful isolation through enzymatic digestion (45), followed by rapid expansion maintaining functional integrity (46), typically achieving 1,000-10,000-fold expansion over 3–6 weeks (47). Current assay platforms encompass functional assessments, phenotypic characterizations, and mechanistic assays (48).

Functional Assays: For both TIL and CAR T-cells, cytotoxicity assays represent cornerstone potency measurements. The classical Chromium-51 release assay remains a gold standard (49), yet Lactate dehydrogenase (LDH) release assays provide safer, colorimetric readouts more amenable to automation (50). Cytokine production assays complement rather than fulfill cytotoxicity measurements. While Interferon-gamma (IFN-y) serves as a standard regulator potency surrogate indicative of T-cell activation, it does not always correlate with direct cytolytic activity. Single-cell analyses demonstrate that cytokine secretion and cytotoxic granule release are regulated through partially independent pathways, uncoupling antigen-specific T cells IFN-γ production from degranulating (51) Hence IFN-y may not fully capture overall “killing” capacity or polyfunctional metrics such as the Polyfunctional Strength Index (PSI), a composite measure of broader T-cell functional quality integrating multiple effector functions (52). Therefore, comprehensive potency assessment requires orthogonal assays measuring both cytokine production and direct cytotoxicity (chromium-51 release, LDH, or impedance-based killing) (53, 54). to capture the functional profile.

### Phenotypic assays

Flow cytometry reveals TIL composition and functional state through immunophenotyping panels (55). Memory T-cell subset distribution provides insights into therapeutic potential, with central memory and stem cell-like memory phenotypes associated with improved persistence. Nonetheless, the relationship between inhibitory receptor expression and T-cell functional capacity exhibits considerable complexity for consideration when interpreting phenotypic potency metrics. Memory T cells with reduced expression of inhibitory receptors including PD-1, T-cell immunoglobulin, mucin-domain containing-3 (TIM-3) and lymphocyte-activation gene 3 (LAG-3) often demonstrate improved functional capacity in certain contexts (56). However, these receptors can also exert alternative context-dependent costimulatory or regulatory functions.

Tim-3, initially characterized as an exhaustion marker, nonetheless exhibits costimulatory properties in specific contexts, promoting effector T-cell responses through Akt/mTOR signaling while simultaneously restricting memory precursor development and mediating resistance to PD-L1 checkpoint blockade during chronic viral infection (57). Tim-3 engagement during antigen stimulation can drive differentiation toward effector memory phenotypes via mTORC1 activation (58). Similarly, PD-1 plays important roles in maintaining proper T-cell proliferation and differentiation kinetics beyond simple inhibition. Complete PD-1 silencing (as opposed to transient antibody blockade) impairs CAR-T proliferation and prevents optimal effector-to-memory differentiation (59), while myeloid-specific but not T-cell-specific PD-1 ablation prevents immunosuppression in certain contexts (60). Under appropriate stimulation conditions, PD-1+ T cells can differentiate back to memory stem cell phenotypes (61), suggesting these cells retain regenerative capacity rather than representing terminally exhausted populations. These findings emphasize that phenotypic characterization of inhibitory receptor expression must be interpreted within the specific therapeutic context, expansion protocol, and timing of assessment. Simple enumeration of PD-1+ or TIM-3+ cells provides limited predictive value without functional correlation; instead, potency assessment should integrate receptor expression patterns with functional readouts and differentiation state markers to comprehensively evaluate therapeutic potential (62).

### CAR T-specific design

CAR T-cells differ fundamentally from conventional T-cells in aspects directly impacting potency assessment requirements. CAR constructs combine antibody antigen-binding domains with intracellular signaling, creating artificial receptors recognizing surface antigens directly without processed peptide major histocompatibility complex (MHC) presentation (8, 63). Design evolution has progressed through multiple generations: First-generation CARs provided cytotoxicity with limited proliferation (64); the second-generation incorporated costimulatory domains (CD28 or 4-1BB), improving expansion and persistence (65, 66); later generations including fifth-generation (5G) CARs built with sophisticated tri-signaling systems require more tailored potency assay approaches (8, 67, 68).

### CAR T-cell potency assays

The standardization of CAR T-cell potency assays has been driven by regulatory requirements for consistent product characterization across different manufacturing platforms. CAR T-cell cytotoxicity assays require specific considerations; *In vitro* functional assessments through target-specific cytotoxicity using co-culture assays (69) involve antigen-positive and antigen-negative control cell lines and assessment across antigen density ranges. Serial killing capacity evaluation tracks T-cell-target cell interactions over extended periods, since single-timepoint cytotoxicity assays may not capture CAR T-cell exhaustion kinetics, recovery capacity, and overall therapeutic potential (70). Activation and proliferation assays measure upregulation of activation markers (CD25, CD69, HLA-DR) and proliferative capacity through CFSE dilution or Ki67 expression (8). Cytokine production assays measure IFN-γ, TNF-α, IL-2, and granzyme B, through ELISA, ELISpot, or intracellular cytokine staining (71). Phenotypic characterization reveals CAR expression levels (8), with presence of central memory and stem cell-like memory T-cells correlating with improved persistence (72). Superior therapeutic outcomes are achieved by manufacturing process that maintain less differentiated T-cell subsets, underscoring differentiation state as a key potency metric (73, 74). Manufacturing-related assays assess transduction success, that is comprehensively evaluated by flow cytometry and PCR, quantifying CAR expression and vector integration, respectively (71). Vector copy number analysis seeks to exclude products with excessive integration that might lead to insertional mutagenesis or aberrant gene expression (8). Residual vector contamination assessment represents a critical safety consideration (75).

### Multi-dimensional potency assays for TIL and CAR T-cells

TIL therapy’s patient-to-patient variability cannot be fully mitigated by standardized manufacturing alone (62), requiring sophisticated potency assays measuring behavior in more physiologically relevant ways (76). Expansion-related changes in cell phenotype require careful monitoring (77) as does tumor specificity verification (78) that can be addressed through synthetic neoantigen libraries (79). Current clinical practice employs several multidimensional TIL potency assays beyond single-parameter measurements. The established reference standard combines IFN-γ quantification (via ELISA, ELISPOT, or intracellular flow cytometry) with assessment of 4-1BB upregulation following tumor cell co-culture, providing integrated readouts of activation and tumor-specific recognition. Recent manufacturing optimization protocols evaluate maintenance of less-differentiated phenotypes through expression of stemness markers (CD62L, CD28, TCF-1) alongside functional cytotoxicity, recognizing that stem cell-like memory states predict superior *in vivo* persistence (80). Integrating 3D technologies described below, advanced TIL characterization platforms employ patient-derived organoids cultured at air-liquid interface, preserving functional TILs with original TCR repertoires and enabling tumor cytotoxicity assessment within architecturally intact structures (81). Organoid-on-chip platforms could compress comprehensive drug response profiling to clinically relevant timelines, facilitating rapid potency-guided therapeutic decision-making (82). These systems assess phenotype, metabolic fitness (mitochondrial biomass) (83), and dual cytotoxic mechanisms (TCR-dependent and NK receptor-mediated killing), particularly for next-generation approaches such as TIL-derived iPSC regeneration technologies (84). Integration of spatial biology enables quantitative mapping of TIL distribution patterns, immune synapse formation, and cytotoxic events, generating multidimensional potency signatures that correlate with clinical outcomes (62).

**Figure 2:**
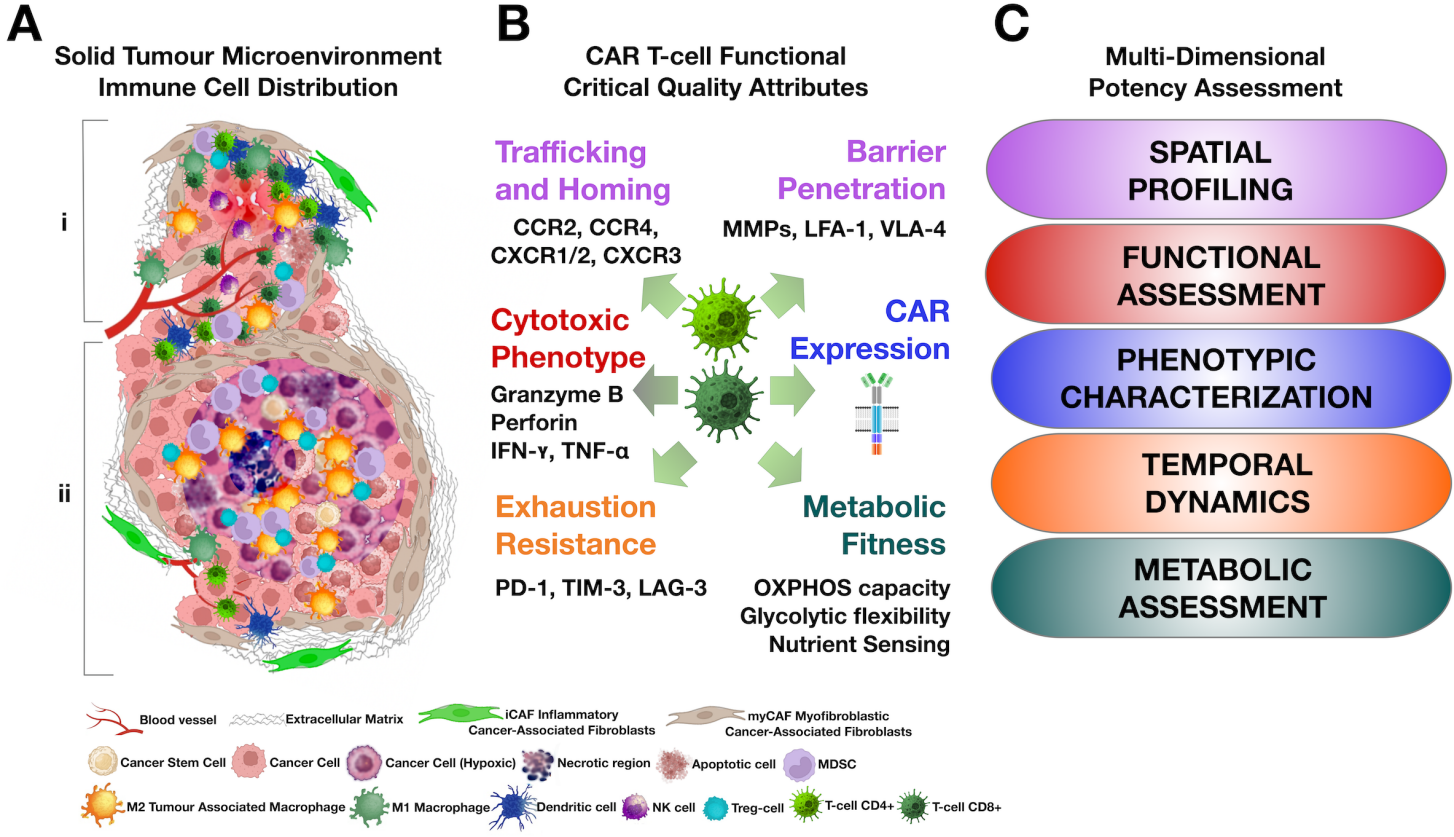
Multi-dimensional potency assessment guided by tumor microenvironment heterogeneity. **(A)** Schematic of solid tumor microenvironment heterogeneity showing distinct spatial regions: region “i” (hot zone) features abundant vasculature allowing infiltration of therapeutic T-cells (TIL and/or CAR T-cells), while region “ii” (cold zone) is characterized by hypoxia, sparse vasculature, macrophage predominance and exclusion of therapeutic T-cells. **(B)** Key functional critical quality attributes for both TIL and CAR T-cell therapies (e.g., trafficking, barrier penetration, cytotoxic phenotype, exhaustion resistance, metabolic fitness). **(C)** Corresponding multi-dimensional assay modalities (spatial profiling, functional assessment, phenotypic characterization, temporal dynamics, metabolic assessment) used to evaluate these attributes across both platforms.

Antigen heterogeneity and microenvironmental barriers leading to antigen escape are formidable obstacles for solid tumor CAR T therapies, that many current potency assays fail to address properly (85). A tumor microenvironment involving hypoxia, nutrient depletion, and immunosuppressive cell populations, fundamentally alters CAR T-cell function (86). Physical barriers, including dense stroma, impede trafficking and tumor infiltration (87). The expression of appropriate trafficking receptors, matrix metalloproteinases, and chemokine receptors require specialized potency assays. Safety considerations include on-target, off- tumor toxicity when target antigens are expressed on healthy tissues. Most critically, metabolic fitness, essential for proliferation and effector function, is usually substantially compromised within solid tumor microenvironments (72, 88). Tumor resistance mechanisms including antigen loss, immune editing, and alternative survival pathway activation, are all factors that complicate potency assessment, with current assays limited in predicting resistance development (88) (**Figure 2**). Despite such challenges, TIL therapy has demonstrated remarkable clinical success in melanoma with recent objective response rates exceeding 50% in pretreated patients (89), while emerging CAR T approaches show clinical activity (26, 90).

## Diversifying CAR-engineered platforms

**Figure 3:**
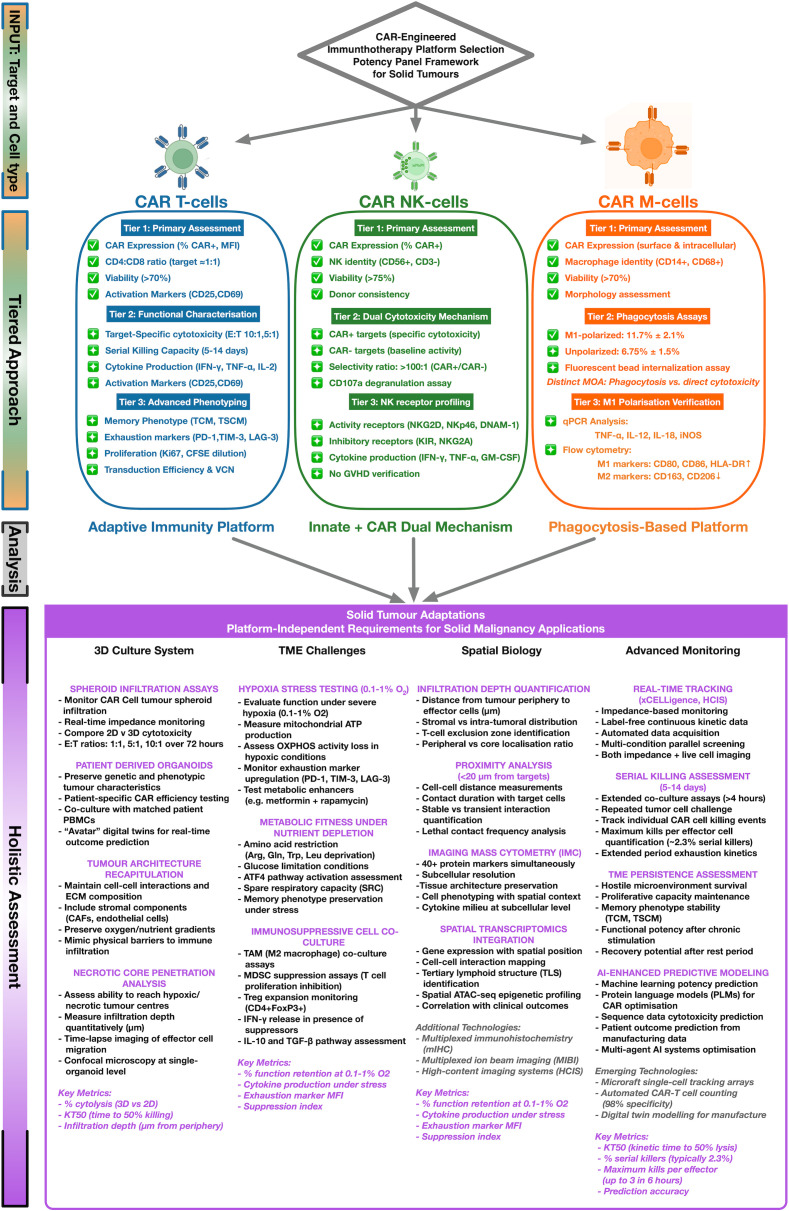
Decision-tree framework for selecting potency assays across CAR-engineered platforms. A summary of assay selection criteria based on mechanism of action (cytotoxicity, degranulation, phagocytosis, cytokine release) and platform-specific quality attributes. Text boxes indicate recommended assay modalities for CAR T, CAR‑NK, CAR‑M, and CAR-γδ T cells; arrows denote iterative refinement loops guided by manufacturing data and clinical correlation. Crucially, while platform- specific assays (e.g., phagocytosis for CAR-M) are essential, core attributes such as serial killing capacity, polyfunctionality, and metabolic fitness represent shared determinants of potency that warrant unified holistic assessment strategies across all modalities.

While CAR T-cell therapy has demonstrated remarkable efficacy in hematologic malignancies, translation to solid tumors remains limited (91, 92). A logical extension that conceptually bridges TIL and CAR T−cell therapies is the emerging strategy of the hybrid CAR−TIL approach, in which TIL are genetically engineered to express CAR constructs, creating a therapeutic population that combines polyclonal tumor recognition with engineered specificity (93). Nonetheless, dedicated potency assay frameworks for such pooled products remain to be developed. Beyond T-cell-based products, diversification of CAR platforms has expanded to innate and unconventional lymphocytes, including CAR-Natural Killer cells (CAR-NK), CAR-macrophages (CAR-M) and CAR-gamma delta (CAR-γδ) T cells (**Table 1**). These platforms offer distinct advantages but require fundamentally different potency assessment strategies that account for their unique killing mechanisms and quality attributes (94) (**Figure 3**).

**Table 1:** Platform-specific potency assays and critical quality attributes across CAR-engineered immunotherapy platforms.

Platform	Primary potency assays	Critical quality attributes (CQAs)	Key regulatory considerations	Maximum technology readiness level	References
TIL (Tumor-Infiltrating Lymphocytes)	Cytotoxicity assays (LDH release, Cr-51 release); IFN-gamma production (ELISA, ELISpot); Tumor-specific cytokine release; Serial killing capacity	Viability and cell count; CD3+ T-cell identity; Memory phenotype (CD45RO+, CD62L+); PD-1/TIM-3/LAG-3 expression; Tumor reactivity; Polyclonality	MOA-linked functional assay required; Patient-to-patient variability necessitates batch-specific testing; Tumor specificity verification recommended; Persistence biomarkers emerging as correlates	TRL 8-9 (FDA-approved product: lifileucel/Amtagvi for melanoma 2024)	(12, 14, 17, 42, 43, 45–50, 55)
CAR T-cells	Target-specific cytotoxicity (co-culture assays with antigen+ and antigen- cell lines); Serial killing capacity; Activation markers (CD25, CD69, HLA-DR); Cytokine production (IFN-gamma, TNF-alpha, IL-2, granzyme B); Proliferation (Ki67, CFSE dilution)	CAR expression level (flow cytometry); Transduction efficiency; Vector copy number; Memory phenotype (central memory, stem-like memory); Exhaustion markers (PD-1, TIM-3, LAG-3); Viability and purity	Standardized cytotoxicity assay essential for comparability; CAR expression quantification mandatory; Persistence and differentiation state correlate with outcomes; Safety: residual vector, insertional mutagenesis risk	TRL 9 (multiple FDA/EMA-approved products: Kymriah, Yescarta, Tecartus, Breyanzi, Abecma, Carvykti)	(8, 56, 63–74)
CAR-NK Cells	Dual cytotoxicity assessment (CAR-dependent and CAR-independent); CD107a degranulation assay; Cytokine/chemokine production (IFN-gamma, TNF-alpha, GM-CSF); NK receptor expression profiling (NKG2D, NKp46, DNAM-1)	CAR expression; NK receptor expression (activating receptors); Viability and expansion fold; Allogeneic donor consistency (if off-the-shelf); Cytotoxic granule content	Must differentiate CAR-mediated from innate NK cytotoxicity; No GVHD risk enables allogeneic manufacturing; Donor-to-donor consistency validation critical for off-the-shelf products	TRL 5-7 (multiple Phase 1/2 clinical trials; no approved products yet)	(94–100)
CAR-Macrophages (CAR-M)	Phagocytosis quantification (dual-fluorescent co-culture, antigen-coated beads); M1 polarization markers (qPCR: TNF-alpha, IL-12, IL-18, iNOS); Immunophenotyping (M1: CD80, CD86, HLA-DR; M2: CD163, CD206); Cytokine profiling	CAR expression; M1 vs M2 polarization status; Phagocytic capacity; Viability and purity; Source cell consistency (monocyte-derived or iPSC-derived)	Potency assessment fundamentally different from T/NK cells (phagocytosis, not cytotoxicity); M1 polarization enhances phagocytosis 7-12-fold; Phase 1 data confirm tumor targeting and safety	TRL 5-6 (Phase 1 clinical trials completed; early Phase 2)	(101–104)
CAR-gamma-delta T-cells	Dual-pathway assessment: 1) CAR-mediated cytotoxicity 2) Phosphoantigen-driven activation (BTN3A pathway); Chemokine receptor expression (CCR5, CXCR3); Rapid cytokine production (IFN-gamma, TNF-alpha); Tissue migration capacity	CAR expression; gamma-delta TCR expression and Vdelta subset distribution; Activation status; Tissue-homing receptor expression; Viability and purity	Must evaluate both CAR-dependent and phosphoantigen-sensing pathways; Synergy between pathways may enhance potency; Preferential tissue migration requires trafficking assays	TRL 4-6 (preclinical and early Phase 1 trials)	(87, 105–107)
*In Vivo* CAR T-cells	Transduction efficiency *in vivo* (PCR, flow cytometry on peripheral blood); CAR expression kinetics post-administration; Functional assessment of *in vivo*-generated CAR T-cells (ex vivo cytotoxicity); Biodistribution (imaging: PET, bioluminescence); Off-target transduction assessment	Vector titer and purity (AAV, LNP-mRNA); *In vivo* transduction efficiency; CAR expression duration; Specificity of targeting (T-cell-specific promoters); Safety: off-target transduction, genotoxicity	Paradigm shift: gene therapy product, not cellular therapy; Potency measured *in vivo* (not ex vivo pre-infusion); Biodistribution and pharmacokinetics critical; Regulatory framework follows gene therapy guidance (FDA S12)	TRL 3-5 (preclinical models; early investigational human studies emerging 2025)	(8, 108–111)

Comparative overview of six major cellular and gene therapy platforms for solid tumor immunotherapy, highlighting primary potency assays, critical quality attributes (CQAs), key regulatory considerations, and technology readiness level (TRL). TIL and CAR T-cells represent mature platforms (TRL 8-9) with approved products and established regulatory precedent. CAR-NK and CAR-M platforms demonstrate distinct potency assessment requirements reflecting unique mechanisms of action (degranulation/innate cytotoxicity for NK; phagocytosis/M1 polarization for macrophages). CAR-gamma-delta T-cells require dual-pathway assessment (CAR-mediated and phosphoantigen-sensing). *In vivo* CAR T-cells represent a paradigm shift toward gene therapy, with potency measured post-administration rather than pre-infusion, necessitating entirely different analytical frameworks aligned with biodistribution and pharmacokinetics rather than ex vivo functional assessment.

### CAR-NK cells

Potency assessment must capture both CAR-dependent and CAR-independent cytotoxicity, as NK cells retain innate tumor recognition capacity through activating receptors (NKG2D, DNAM-1, NKp46) that recognize stress-induced ligands independent of CAR engagement (94, 95). Standard CD107a degranulation assays measure lysosome mobilization indicating cytotoxic granule release, performed by co-culturing CAR-NK cells with target cells in the presence of anti-CD107a antibody and analyzing CD107a positive NK cell percentages by flow cytometry (95, 96). For CAR-NK products, degranulation must be measured against both CAR-positive and CAR-negative target cell lines to differentiate CAR-mediated killing from baseline natural cytotoxicity (94). Dual cytotoxicity mechanisms require evaluating NK receptor expression profiles (NKG2D, NKp46, DNAM-1) alongside CAR expression, as both contribute to therapeutic potency. Unlike CAR T cells, CAR-NK cells do not cause graft-versus-host disease, a trait that can be enhanced (97) to enable allogeneic “off-the-shelf” manufacturing from healthy donors, cord blood, or iPSC-derived sources. This manufacturing advantage requires donor-to-donor consistency validation through standardized degranulation and cytokine production assays (98).

### CAR-macrophages

CAR-M potency assessment differs fundamentally from CAR T and CAR-NK approaches, focusing on phagocytosis and M1 polarization rather than direct cytotoxicity (101, 102). Phagocytosis is quantified using dual-fluorescent co-cultures: fluorescently labeled tumor cells (mCherry) engulfed by GFP-expressing CAR-M cells produce double-positive signals. M1-polarized CAR-M cells show significantly enhanced phagocytosis (6.75% to 11.7%) compared to unpolarized cells (102). Alternative methods employ fluorescent antigen-coated beads that fluoresce only upon internalization and acidification, enabling real-time monitoring (101). M1 polarization verification requires qPCR confirmation of activation genes (TNF-α, IL-12, IL-18, iNOS) and immunophenotyping (M1 markers: CD80, CD86, HLA-DR; M2 markers: CD163, CD206) (103). Phase 1 clinical data confirmed CAR-macrophages effectively target tumors with immune activation and acceptable safety, validating this distinct potency assessment approach (103).

### CAR-gamma delta T cells

CAR-γδ T cells require specialized potency assessment because they recognize antigens through two distinct pathways (105, 112); CAR-mediated targeting and intrinsic phosphoantigen sensing independent of MHC-peptide presentation. The phosphoantigen pathway involves BTN3A proteins on tumor cells interacting with natural gamma delta T-cell receptors; BTN3A activation occurs when tumor cell phosphoantigens induce conformational changes (112). Potency assessment must evaluate (1): CAR-mediated tumor cell killing (2), phosphoantigen-driven activation using mevalonate inhibitor-treated tumor cells that enhance phosphoantigen production, and (3) synergy between both pathways (113). Selective pathway blockade clarifies each contribution. Importantly, gamma delta T cells preferentially migrate to tissues and rapidly produce immune cytokines (IFN-γ, TNF-α) upon activation (105), requiring assessment of chemokine receptor expression (CCR5, CXCR3) and cytokine production as functional potency measures beyond standard killing assays.

## New approach methodologies in CAR T-cell analytics

New Approach Methodologies (NAMs) encompass a broad suite of innovative *in vitro*, in silico and integrated data−driven approaches designed to generate human−relevant, mechanistic information and, where possible, to reduce reliance on traditional animal−based assays. In the context of CAR−T cell analytics, NAMs include advanced 3D culture systems, microphysiological platforms, spatially resolved omics, high−content imaging, and AI−enabled computational models that can refine our understanding of potency, durability and safety. Emerging safety−focused NAMs also include hiPSC−derived normal cell panels combined with impedance−based or high−content readouts to evaluate on−/off−target cytotoxicity and organ−specific risk for CAR−T candidates (114). Conceptually and pragmatically, it is useful to distinguish between currently validated GMP lot−release assays and NAM−type platforms.

The latter have not yet been demonstrated to be suitable as stand−alone release assays in routine GMP lot−release settings and are at earlier stages of qualification. At present, they primarily inform process development, mechanistic characterization and candidate critical quality attribute (CQA) discovery, with a view to potential future integration into formal release testing as their performance, robustness and regulatory experience mature. In contrast, GMP−viable technologies for release testing are currently limited to flow−cytometry–based immunophenotyping and CAR expression quantification, validated ELISA platforms for cytokine quantification, chromium−51 or LDH−based cytotoxicity assays with established acceptance criteria, and real−time impedance−based cytotoxicity platforms (for example, xCELLigence) that are nearing late−stage validation (8). These assays have met stringent ICH Q2(R2) expectations for accuracy, precision, robustness, and inter−laboratory transferability and are therefore appropriate for lot−release decisions.

### Integrating 3D culture, spatial biology and advanced imaging

Cell monolayer-based assays fundamentally fail to recapitulate the spatial organization and microenvironmental heterogeneity defining solid tumor biology (115, 116). Within the NAM framework, 3D systems have increasingly been adopted to address these limitations. Spheroid models self-assembled under non-adherent conditions can recapitulate some key features of solid tumors including proliferative rims, quiescent intermediate zones and necrotic cores (117), enabling co-culture assays that captured T-cell infiltration dynamics and resistance gradients not observed in 2D monolayers (118). Relationships between spheroid size, cellular density and therapeutic efficacy, as well as hypoxia-dependent changes in CAR T-cell function and immune resistance gradients have thus been explored primarily in such 3D systems (119, 120). Patient−derived tumor organoids (PDTO) have offered additional opportunities for personalized potency assessment, maintaining many genetic, phenotypic and architectural features of the source tumor while remaining experimentally tractable (121, 122). Reported establishment success rates exceeded 50% for many solid tumor types but can vary widely by indication and sample type, with high rates in glioblastoma (~91%) (123), breast (>80%) (124) and colorectal cancer (76-93%) (125), but substantially lower rates in prostate cancer (10-16%) (126, 127) and some heavily pre−treated lesions. A persistent practical limitation has been the temporal mismatch between PDTO generation (typically 2–4 weeks to reach assay−ready cultures) (123) and accelerated CAR−T manufacturing timelines of 7–14 days (128). Facing the challenges of pre-emptive organoid banking from diagnostic biopsies, organoid data has predominantly been useful to inform late−stage characterization, comparability and future optimization rather than real−time autologous release decisions. Additional constraints, including batch−to−batch variability, reliance on animal−derived matrices and specialized technical infrastructure, have further kept organoids in research−stage NAM level.

Although organoid models are generally recognized as more cost-effective than patient-derived xenograft (PDX) models and can be generated more promptly (129), costs per established organoid line remain substantial and vary considerably depending on tumor type, culture duration, throughput scale, and extent of molecular characterization required. In addition, uncertain success rates and relatively prolonged generation timelines, have required careful cost-benefit considerations for each application involving time-sensitive clinical manufacturing decisions in autologous cell therapy contexts (130). These economic and logistical constraints, together with intrinsically non-vascularized architecture of most current organoids (leading to poorly representative diffusion-limited gradients) and specialized infrastructure requirements, have further limited the use of PDTOs to research-stage NAM applications rather than routine batch release. Nonetheless, organoids may uniquely preserve a biological fidelity of tumor-resident immune populations and stromal architecture that may be key for overcoming *in situ* immunological barriers but lacking in simpler tumor models (131). Ongoing work on automated culture systems, microfluidic organ−on−chip formats and synthetic matrices has begun to improve standardization and throughput (132, 133). Current consensus favors a dual strategy: advancing standardization, microfluidic platforms, and automation to enhance organoid feasibility for mechanistic potency assessments, while developing validated surrogate assays and biomarker panels for routine lot release (134).

As a viable compromise, when organoid derivation from the patient is not possible, models representing diverse solid tumor types, including colorectal, pancreatic, lung, and brain cancers (123), can retaining tumor-specific characteristics relevant to an immunotherapy response (135). Integration of both spheroid and organoid approaches within potency testing workflows establishes comprehensive evaluation from standardized quality control (QC) to individualized therapeutic optimization (118).

Recent advances have improved isolation protocols, automated culture systems, and standardized media formulations (136). Organ-on-chip platforms have achieved real-time monitoring and precise control over cellular microenvironments while maintaining organoid complexity (137). Integration of automated imaging and T-cell infiltration tracking pattern analysis has allowed high-content screening approaches that can quantify cytotoxic activity with single-cell resolution within complex 3D structures (138). Avoiding batch-to-batch variability and potential confounding factors from Matrigel and similar animal-derived matrices (139), synthetic and semi-synthetic hydrogel-based systems using polyethylene glycol (PEG), alginate or synthetic peptide sequences can provide improved standardization with controlled tuning of mechanical properties, degradability, and cell adhesion characteristics (140). Incorporated bioactive molecules and degradable linkages allow T-cells to remodel their microenvironment during infiltration (141). Emerging matrix-free 3D culture approaches using magnetic bioprinting, acoustic assembly and specialized culture geometries may further help standardization, reproducibility and cost-effectiveness (142).

There is need to test potency within physiologically relevant oxygen gradients, since T-cell functionality undergoes significant modifications under hypoxic conditions. Solid tumors characteristically exhibit steep oxygen gradients; well-oxygenated regions near blood vessels transitioning to severely hypoxic areas in poorly perfused regions (143). This has been mimicked through specialized incubators with microfluidic devices bearing oxygen gradients (144, 145). An emerging clinostat technology with ultra-low shear forces has proven adept at maintaining physiologically representative metabolic capacity and transcriptional profiles (146, 147). Current hypoxia modules enable precise oxygen control from atmospheric to physiological levels that can drop to 0.1% within tumor regions, substantially altering T-cell functionality (148). Low oxygen directly inhibits T-cell proliferation, reduces cytotoxic granule production, and shifts cytokine secretion toward immunosuppressive profiles (149). Hypoxia also promotes accumulation of regulatory T-cells and myeloid-derived suppressor cells (150, 151). Beyond direct oxygen limitation, metabolic consequences include altered glucose utilization, lactate secretion, and ATP generation; creating metabolic competition that limits T-cell nutrition (152).

Spatial biology has fundamentally transformed understanding of cellular immunotherapy mechanisms and potency assessment (153, 154), enabling resolution of heterogeneity and trafficking quantification (155). Physical positioning of therapeutic cells relative to targets, support structures, and inhibitory elements directly influences outcomes (156). This revolution rests on three technological advances: multiplexed imaging, single-cell sequencing with spatial preservation, and computational methods for analyzing complex spatial datasets (157).

High-Content Imaging Systems (HCIS) could capture complex cellular behaviors across spatial and temporal dimensions (158). For immunotherapy potency assessment, HCIS enabled simultaneous multi-parameter assessment of therapeutic cell migration, target cell recognition, immune synapse formation, and cytotoxic events with single-cell resolution. Such analysis capabilities have paved the way for simultaneous assessment of dozens of cellular features, including morphological characteristics, protein expression patterns, metabolic states, and functional activities (159). Time-lapse imaging capabilities add temporal resolution, enabling tracking of dynamic processes such as T-cell activation kinetics, serial killing capacity and persistence under stress conditions (160). Artificial intelligence and machine learning algorithms have transformed data analysis capabilities, automating identification of complex cellular behaviors and interaction patterns (161). Hence, subtle phenotypic signatures associated with optimal potency measurement, leading to better prediction of clinical performance, may emerge from hitherto ignored *in vitro* observations.

Imaging Mass Cytometry (IMC) could detect over 40 proteins simultaneously while preserving tissue architecture (162). For potency testing, IMC assesses therapeutic cell phenotypes, activation states, target recognition, and microenvironmental interactions within complex 3D tumor environments (162−165). Multiplexed immunofluorescence approaches enable detection of 10–60 proteins simultaneously through sequential staining and imaging cycles, creating comprehensive protein expression maps with spatial information (163). Proximity-based assays, enable detection of protein-protein interactions and cellular contacts with high spatial resolution (164), directly measuring immune synapse formation (165).

Integrating single-cell RNA sequencing (ssRNAseq) with spatial preservation highlights immunotherapy mechanisms at molecular resolution within the tissue context (166, 167). Technologies such as Visium, MERFISH, and seqFISH+ measure gene expression patterns across thousands of cells while preserving their spatial coordinates (168). Spatial transcriptomics can reveal how therapeutic cells adapt to different microenvironmental conditions, identify factors that promote or inhibit therapeutic function and map the molecular basis of spatial heterogeneity in therapeutic responses (169, 170). Advanced computational methods enable reconstruction of cellular communication networks, identification of ligand-receptor interactions, and mapping of signaling pathways within specific spatial domains (171). Spatial statistics and computational geometry enable quantitative analysis of therapeutic cell distribution patterns, revealing organizational principles governing therapeutic outcomes (175−177). Moreover, machine learning approaches can identify spatial patterns associated with enhanced therapeutic efficacy (172) providing predictive models that guide optimization (173). Such insights provide foundations for streamlined assays that capture the most predictive aspects of therapeutic cell function (8). Machine learning approaches applied to comprehensive spatial datasets can identify minimal parameter sets that retain maximal predictive power, fostering simplified cost-effective assays (174, 175).

### Label-free and real-time monitoring approaches

Traditional labeled detection using fluorescent labels (176), radioactive tracers or enzyme-linked detection systems risk introducing significant perturbations in cellular physiology through multiple mechanisms (177), compromising accuracy and physiological relevance. Even subtle changes in T-cell activation, migration, and cytotoxic capacity can have significant outcomes, making label-induced perturbations important. Photobleaching can induce cellular damage at surprisingly low light doses (178). Endpoint measurement strategies inherent in many labeled assays provide only snapshots of dynamic biological processes, missing critical temporal information of particular significance for immunotherapy potency assessment (179).

As one of several notable examples (**Table 2**), the xCELLigence Real-Time Cell Analysis (RTCA) platform measures cellular impedance to non-invasively monitor surface-tethered tumor cell viability in the presence of immune effector cells without labels (186). Real time quantitative parameters such as KT50 (Time to Kill 50% of the target tumor cells) and cytolysis percentages, provide complementary metrics for preclinical potency tests, accelerating assay workflows (187). Atomic force microscopy (AFM) provides nanomechanical characterization at high resolution (180, 188). Mechanotransduction pathways critically regulate CAR T signaling with binding forces contributing to T-cell effectiveness and potency (181, 185). AFM revealed a negative correlation between the binding forces of next generation dual-target CAR T-cells with antigen and subsequent CAR T-cell efficacy in animal experiments (181). This apparent paradox is nonetheless consistent with the idea that weaker interactions can lead to faster detachment from dying cells, allowing more effective “serial killing” of multiple target cells in succession to enhance tumor clearance (185). Machine learning strategies are being adopted to enhance instrumentation and data interpretation (189).

**Table 2:** Comparison of emerging label-free technologies.

Technology	Principle	Key advantages	Primary application	Limitations	Maximum TRL	tr status	GMP suitability	Regulatory precedent	Cost/sample	Throughput	References
Impedance Monitoring (xCELLigence RTCA)	Electrical impedance monitoring of adherent target cell viability	Continuous real-time monitoring; KT50 quantification; no labels required; automated data acquisition	CAR T-cell cytotoxicity against adherent targets; serial killing assessment; release testing candidate	Requires adherent target cells; limited to 2D culture; target cell density critical	7-8	Qualified for exploratory use; under validation for release testing	High (automation-ready, GMP-compatible instrumentation)	Used in IND submissions; no FDA-approved product precedent	Moderate	High (96-well format, 50–100 samples/day)	(176, 177)
Atomic Force Microscopy (AFM)	Nanoscale mechanical force measurement at single-molecule resolution	High-resolution mechanical mapping; direct binding force measurement; nanometer-scale precision	CAR T-cell binding affinity characterization; mechanotransduction studies; advanced research	Low throughput: specialized expertise required; time-intensive; expensive equipment	4-5	Exploratory research stage; no validation for GMP	Low (manual operation, research-grade instrument)	No GMP or regulatory precedent; preclinical research only	Very High	Very Low (<5 samples/day)	(178, 180, 181)
Digital Holographic Microscopy (DHM)	Quantitative phase imaging without labels; time-resolved 3D visualization	Label-free 3D tracking; quantitative metrics (volume, dry mass); non-invasive	Cell morphology assessment; single-cell tracking in 3D; migration dynamics	Complex data processing; specialized software; requires transparent samples	5-6	Qualified for exploratory characterization	Moderate (semi-automated, requires expertise)	No GMP precedent; used in research publications	High	Moderate (20–40 samples/day)	(182, 183)
Optical Metabolic Imaging (OMI)	Autofluorescence of metabolic coenzymes (NADPH, FAD) without exogenous labels	Metabolic fitness assessment; single-cell resolution; non-invasive; real-time	Manufacturing QC; potency prediction; metabolic characterization during culture	Specialized equipment: validation needed; limited clinical correlation data	6-7	Under qualification for manufacturing QC	Moderate to High (automation feasible with dedicated system)	No regulatory precedent; emerging in academic/industry studies	High	Moderate (30–60 samples/day with automation)	(184)
VivaCyte Platform (CellPly)	Optical imaging without fluorescent labels; continuous single-cell tracking	Single-cell resolution; serial killing quantification; identifies serial killer phenotypes	T-cell cytotoxicity assessment; sequential killing dynamics; functional profiling	Currently 2D microwell-based only; limited 3D capability; proprietary platform	6-7	Qualified for exploratory functional assays	Moderate (microwell format, semi-automated)	No GMP precedent; used in TCR validation studies	Moderate to High	Moderate to High (48–96 well, 30–70 samples/day)	(185)

Comparison of emerging label-free technologies for potency assessment. Overview of five representative label-free technologies with expanded characterization including technology readiness level (TRL), validation status, GMP suitability, regulatory precedent, estimated cost per sample, and throughput capacity. Technologies span electrical impedance (xCELLigence RTCA), nanomechanical force measurement (AFM), quantitative phase imaging (DHM), Metabolic Imaging (OMI), and optical single-cell tracking (VivaCyte platform). TRL scores reflect maturity from exploratory research (TRL 4, 5), through pilot validation (TRL 6, 7), to near-GMP-ready (TRL 7, 8). While xCELLigence RTCA demonstrates highest GMP suitability and has been used in IND submissions, other platforms remain primarily in exploratory or qualification stages, highlighting the gap between analytical sophistication and regulatory readiness for release testing. TRL scoring adopts EU Horizon Europe definitions: TRL 1–3 = basic principles to experimental proof of concept; TRL 4–6 = technology validated/demonstrated in lab or relevant (pilot) environment; TRL 7–9 = system prototype to fully qualified system in operational environment. Cost/sample categories use approximate euro ranges to reflect typical EU pricing (Low <€100; Moderate €100–€500; High €500–€2000; Very high >€2000). Throughput estimates assume dedicated operator and optimized protocols. Validation status reflects the current situation in 2026 and may change as the regulatory framework evolves.

Digital Holographic Microscopy (DHM) provides time-resolved quantitative phase imaging (QPI) (190), enabling 3D visualization and label-free analysis of living cells (179). Digital in-line holographic microscopy (DIHM) is simpler and more robust for cell tracking and identification in relatively large volumes (179). Optical Metabolic Imaging (OMI) is a label-free technique to monitor the metabolic fitness of CAR T-cells during manufacture. Autofluorescence intensities and lifetimes of metabolic coenzymes like NAD(P)H and FAD are used to assess real-time metabolic shifts linked to T-cell activation. OMI revealed that culture media composition (e.g., ImmunoCult XF vs. TexMACS) directly influenced oxidative metabolism and cell persistence, altering metabolic kinetics and therapeutic outcomes; identifying parameters correlated to potency (184). The CellPly VivaCyte platform utilizes optical imaging without fluorescent labels and has helped validate machine learning approaches for identifying tumor-reactive T cell receptors from single-cell RNA sequencing data (191). According to company technical documentation, continuous real-time monitoring of individual cellular interactions and precise quantification of sequential killing events can identify T-cell subsets with “serial killer” phenotypes. However, currently, analysis is restricted to cell monolayer microwell-based culture platforms, constrained in fully recapitulating 3D solid tumor microenvironments.

Real-time systems capture transient events and rapid response kinetics missed by endpoint approaches. Continuous data acquisition has revealed previously unrecognized temporal patterns correlating with therapeutic efficacy (192, 193). This is particularly important for detecting cytotoxicity when the onset of killing might be delayed due to low Effector: Target ratios or low-affinity T-cell interactions (187). The sensitivity of real-time monitoring to subtle changes in cellular behavior has enabled more stringent QC standards and faster manufacturing decisions (194). Real-time assays can more cost-effectively detect batch-to-batch variations before endpoint assays, flagging suboptimal products before extensive resources are committed. Nonetheless, standardized real-time monitoring protocols require robust validation to demonstrate equivalent performance to traditional endpoint assays (195). A shift toward ultra-rapid CAR T-cell manufacturing (196) (e.g. ≤3 days) and emerging non-viral delivery methods expose inadequacies of conventional potency markers, highlighting need for biomarkers more suited to adaptive QC frameworks.

## Artificial intelligence and machine learning integration

AI-powered platforms are now capable of identifying subtle cellular phenotype patterns that remain elusive to conventional analytical methods. Machine learning algorithms can assist immunotarget discovery (197) yet moreover, analyze complex imaging datasets to automatically identify cellular phenotypes associated with enhanced potency, reducing both analysis time and subjective interpretation bias (198, 199). Deep learning automates imaging analysis (200), with neural networks enabling robust image segmentation. These networks can simultaneously segment and identify different mammalian cell types grown in co-culture, with quantitative comparison demonstrating improved accuracy and speed (201). AI can reveal previously unrecognized biomarkers (202), with natural language processing integrating literature knowledge (203, 204). Clinical data-driven deep learning methods have enabled development of automated high-throughput microfluidic platforms simultaneously tracking T cell infiltration and cytotoxicity in solid tumor models (205). AI-informed computation of structural and biophysical feature analysis, was able to enhance CAR design, improving fitness scores for therapeutic potency against solid tumor targets. Computationally optimized tandem CARs cleared tumors in 4 out of 5 pediatric brain tumor models, significantly outperforming prevalent single-targeted CARs that uniformly failed (206). AI-based tools for CAR structure optimization (207) and automated cytotoxicity assessment platforms can more systematically identify key parameters for cytokine production, target cell lysis, and immune cell persistence.

### Supervised learning

This has allowed development of remarkably predictive models for immunotherapy potency based on multiplex cellular characterization data (208), with Transformer modules achieving substantial improvements in processing sequential data (209). Transfer learning enables model adaptation (210) so potency prediction models can be adapted to new therapeutic modalities with minimal additional training data, reducing requirements for new assay development.

### Unsupervised learning

This has revealed hidden structure in immunotherapy potency datasets. The CytoCommunity algorithm could identify tissue cellular neighborhoods based on cell phenotypes and spatial distributions, map learning directly from the cell phenotype space using a graph neural network (GNN) model (211). GNNs could identify complex spatial patterns not apparent through traditional analytical approaches and predict tissue properties based on cellular organization (212, 213). Multi-Omic Synthetic Augmentation (MOSA), a specifically designed unsupervised deep learning model, successfully generated molecular and phenotypic profiles while increasing the efficiency of cell clustering (214), being valuable for biomarker discovery (215) and flow cytometry surveillance (216). Anomaly detection could identify manufacturing deviations including morphological changes in T-cells transduced to express CAR protein (217).

### Multi-modal integration enables comprehensive characterization

Recent advances in multi-modal machine learning have enabled the development of models that can simultaneously analyze imaging data, flow cytometry profiles, genomic information, and functional assay results to generate integrated potency assessments (218). Graph neural networks excel at modeling complex relationships between cellular phenotypes, functional properties, and manufacturing parameters (211). Attention mechanisms can identify the data modalities most relevant for specific predictions (219). The fusion of real-time monitoring data with endpoint measurements enables AI models to capture both dynamic and static aspects of therapeutic cell behavior, enabling more nuanced evaluation for QC cross-validation (220). Digital twins represent an emerging in silico NAM, integrating multi−scale biological and clinical data into virtual replicas of patients or manufacturing processes to enable scenario testing and optimization without additional animal or patient exposure (221). The “digital twin” concept offers transformative potential for advanced therapy quality control (222). Digital twins can avoid reliance solely on terminal potency assays by synthesizing multi-dimensional datasets spanning donor cell characteristics, expansion kinetics, phenotypic drift, functional assay outputs, and environmental parameters across the entire manufacturing workflow, enabling informed and timely release decisions. Beyond quality control, digital twin frameworks are increasingly relevant for bioprocess design, donor tissue selection, and patient stratification (223). Full realization will require integrated software platforms, machine learning-enabled applications, and cross-sector collaboration to establish accessible, validated tools beyond current prototype systems. Highlighting where development is needed for routine deployment, **Table 3** presents technology readiness assessments for key emerging potency assay technologies.

**Table 3:** Technology readiness assessment for emerging potency assay technologies.

Technology	Description	Primary application	Maximum TRL	GMP feasibility	Regulatory status	Validation challenges	References
Impedance Monitoring (xCELLigence RTCA)	Real-time, label-free electrical impedance measurement monitoring target cell viability during immune effector co-culture	Serial killing quantification; KT50 determination; release testing candidate for CAR T cytotoxicity	7-8	High (automation-ready, GMP-compatible instrumentation, 96-well format)	Used in IND submissions; no FDA-approved product precedent yet	Requires adherent target cells; standardization of E:T ratios; acceptance criteria establishment	(54, 186, 194)
3D Tumor Spheroid Models	Self-assembled multicellular tumor structures recapitulating necrotic cores, proliferating outer layers, and oxygen/nutrient gradients	Exploratory characterization of T-cell infiltration dynamics; barrier penetration assessment	5-6	Moderate (manual culture, batch variability, Matrigel dependence)	No GMP precedent; research-stage exploratory tool	Standardization across labs; batch-to-batch spheroid variability; time-intensive (5–14 days)	(115–117, 119, 120, 158, 182, 224)
Patient-Derived Tumor Organoids (PDTOs)	3D cultures maintaining genetic, phenotypic, and architectural features of original patient tumors	Personalized potency testing; autologous therapy optimization; post-manufacturing characterization	4-6	Low to Moderate (establishment success 50-90% tumor-dependent; 2–4 week generation time)	No GMP precedent; exploratory research and personalized medicine context	Temporal mismatch with CAR T manufacturing (7–14 days); high cost; success rate variability	(81, 118, 122–127, 129, 131, 135–139, 225–227)
Spatial Transcriptomics (Visium, MERFISH, seqFISH)	Gene expression profiling with spatial coordinate preservation; maps molecular states within tissue architecture	Exploratory mechanistic studies; identifying therapeutic cell adaptation to microenvironments; advanced characterization	4-5	Low (research-grade, high cost, specialized expertise, complex bioinformatics)	No GMP or regulatory precedent; pure research tool	High cost per sample ($1000-5000+); complex data analysis; no established acceptance criteria	(164, 166–168, 228–232)
Imaging Mass Cytometry (IMC)	Multiplexed detection of >40 proteins simultaneously while preserving spatial tissue architecture	Exploratory spatial profiling of therapeutic cell phenotypes, activation states, and microenvironmental interactions	5-6	Low to Moderate (specialized instrument, tissue section preparation, complex analysis)	No GMP precedent; research and advanced characterization	High equipment cost (>$1M); requires tissue sections; throughput limited; expert interpretation needed	(157, 159, 162, 228, 229, 233, 234)
Optical Metabolic Imaging (OMI)	Label-free autofluorescence imaging of metabolic coenzymes (NADPH, FAD) to assess cellular metabolic fitness	Manufacturing QC; metabolic fitness biomarker; potency prediction during culture	6-7	Moderate to High (automation feasible, specialized equipment required, validation underway)	No regulatory precedent; emerging in industry QC workflows	Validation needed; limited clinical correlation data; specialized equipment ($200K-500K)	(184)
AI-Powered Image Analysis (Deep Learning)	Automated cellular phenotype recognition, interaction pattern identification, and potency prediction from imaging datasets	High-throughput functional screening; automated quality assessment; subtle phenotype pattern discovery	6-7	Moderate to High (cloud-based platforms emerging, FDA guidance on AI released 2024)	FDA guidance emphasizes transparency (2024); no approved AI potency assay yet	Algorithm validation; explainability (XAI) requirements; training data needs; regulatory acceptance path undefined	(50, 158, 197–202, 205, 206, 217, 235, 236)
Digital Holographic Microscopy (DHM)	Quantitative phase imaging providing label-free 3D visualization and tracking of living cells	Cell morphology assessment; single-cell migration tracking; dynamic behavior quantification	5-6	Moderate (semi-automated, requires transparent samples, specialized software)	No GMP precedent; research publications only	Complex data processing; specialized expertise; limited throughput; validation needed	(182, 183, 190, 237, 238)
Atomic Force Microscopy (AFM)	Nanoscale mechanical force measurement for characterizing CAR T-cell binding forces to target antigens	Advanced mechanistic characterization; binding affinity assessment; exploratory research	4-5	Low (manual operation, highly specialized, low throughput, research-grade only)	No GMP or regulatory precedent; pure research tool	Very low throughput (<5 samples/day); expensive; requires specialized training; no path to release testing	(180, 181, 188, 189, 239)
High-Content Imaging Systems (HCIS)	Automated multi-parameter imaging capturing cellular behaviors across spatial and temporal dimensions	Multi-parameter potency assessment; immune synapse formation; serial killing dynamics; time-lapse functional assays	6-7	Moderate to High (automation-ready platforms available, requires validation for GMP context)	No GMP release testing precedent; used in characterization studies	Complex image analysis; large data storage needs; standardization of analysis pipelines	(153, 155–159)
Hypoxia-Controlled 3D Culture Systems	Microfluidic or specialized incubators enabling precise oxygen gradient control (0.1%-21% O2) mimicking tumor physiology	Exploratory assessment of T-cell function under physiologically relevant hypoxia; microenvironmental stress testing	5-6	Low to Moderate (specialized equipment, complex setup, manual operation)	No GMP precedent; research-stage exploratory tool	Standardization of oxygen gradients; integration with other assays; time-intensive; cost	(142–146, 179, 240–244)
Digital Twin Technology (In Silico Manufacturing Models)	Virtual replica of manufacturing process integrating real-time data for predictive process control and potency monitoring	Process optimization; predictive analytics; real-time quality monitoring; personalized therapy design	4-5	Moderate (cloud-based platforms feasible, requires extensive training data, regulatory framework emerging)	FDA discussion stage (2024); no approved implementation for potency assurance	Requires vast training datasets; model validation; regulatory acceptance path undefined; data governance	(222, 245–248)

Comprehensive evaluation of twelve emerging analytical technologies for immunotherapy potency assessment, categorized by technology readiness level (TRL), GMP feasibility, regulatory status, and validation challenges. Technologies range from near-GMP-ready (TRL 7-8: xCELLigence RTCA, AI-powered image analysis, OMI) through pilot validation (TRL 5-7: 3D spheroids, HCIS, spatial transcriptomics) to exploratory research stage (TRL 4-5: AFM, digital twins). GMP feasibility spans from high (automation-ready impedance monitoring, AI-enabled platforms) to low (specialized research-grade instruments like AFM and spatial transcriptomics). This assessment captures both the analytical strengths and key limitations of each platform (standardization, throughput, cost, regulatory validation), highlighting the distinction between technologies realistic for near-term GMP lot-release quality-control testing and exploratory or research-stage NAMs for advanced characterization.

### Currently validated or late−stage applications (TRL 6–9)

Machine−learning tools have already been used in potency−adjacent settings such as image analysis and flow−cytometry automation, where deep learning models can match or exceed expert performance while reducing inter−operator variability and analysis time. Unsupervised algorithms for high−dimensional cytometry (e.g. FlowSOM, Phenograph, UMAP−based clustering) (249) have been increasingly adopted for process characterization and lot−to−lot comparability in complex CAR T-cell products, even though they have not yet been regarded as primary release assays (250). Supervised models combining manufacturing parameters, phenotypic features and outcomes have begun to identify composite signatures associated with clinical response, informing candidate CQAs for prospective validation rather than replacing conventional potency assays (251).

### Developmental applications (TRL 4–7)

Emerging AI tools have begun to analyze real−time culture images and online sensor data to flag process deviations earlier than traditional QC timepoints (252). Machine-learning methods integrating multi-omic data sets have generated richer, more predictive profiles of potency-relevant CAR T-cell product phenotypes (217). These approaches have also shown promise for process understanding and exploratory biomarker discovery (253), but current regulatory frameworks do not yet support their use for real−time release or autonomous decision−making because of concerns surrounding model interpretability, validation under change control, and robustness across sites and populations (254).

### Conceptual frameworks and digital twins (TRL 2–5)

Digital twin concepts for CAR technologies (virtual replicas of patients or manufacturing processes that integrate multi−scale biological and clinical data) remain largely research tools, with exploratory applications in oncology but no near−term role as qualified potency assays. Substantial barriers include incomplete mechanistic understanding of tumor–immune dynamics, the need for extensive longitudinal datasets for calibration and validation, and the absence of regulatory pathways for simulation−based product release or treatment adaptation (255).

### Translational integration and clinical correlation (TRL 3–8)

Translational integration focuses on relating ex vivo potency readouts and multidimensional product data to exploratory pharmacodynamic biomarkers, clinical responses and real-world outcomes across the product lifecycle; to explore which assay patterns are most informative for risk-stratification and treatment optimization. In CAR T−cell therapy, multi−omic signatures derived from the product, the patient and the tumor microenvironment are increasingly used to discover and refine composite biomarkers that predict expansion, persistence, toxicity and durable remission, moving beyond single−analyte correlates (256, 257). These signatures can then be locked into targeted panels and embedded within manufacturing analytics or post−infusion monitoring strategies, where they complement core potency assays by informing dose selection, patient stratification and adaptive risk management, while remaining subject to rigorous analytical validation and regulatory scrutiny. Over time, integration of longitudinal pharmacodynamic data, electronic health−record–derived outcomes and digital manufacturing intelligence is expected to support iterative updating of these translational models, but current use remains predominantly supportive and exploratory rather than a standalone basis for licensure−level potency decisions (258).

### Regulatory and data−governance considerations

Across these AI/ML maturity tiers, from conceptual digital twins to developmental applications, agencies currently position AI/ML as supportive tools that augment human judgment within a risk−based GMP framework, rather than autonomous systems for potency determination. FDA guidance emphasizes algorithm transparency (259) and explainable AI (XAI) focuses on understanding how a model arrives at its predictions before those outputs can influence regulated decisions. The prospect that saliency−based methods and related XAI techniques accurately highlight which input features drive predictions becomes increasingly critical for AI systems designed to support rather than replace human expertise in evaluation. Saliency maps can indicate aspects of the input that most contribute to the model’s prediction (260). Although saliency maps are being explored as predictive biomarkers for treatment response (40), their use to prioritize biomarkers for potency assays in solid cancer immunotherapy products remains under-explored. Cloud−based and federated−learning platforms enable collaborative model development with a minimal local computational burden and a reduced need to centralize raw patient−level data (261). However, to ensure secure, auditable data flows from analytical instruments into AI models and back into QC actions, these platforms must be integrated with existing laboratory information management systems (LIMS) and manufacturing execution systems (MES) (262). Standardization of AI model validation procedures and performance metrics is increasingly recognized as essential for reproducible implementation across organizations and regulatory jurisdictions (263). Looking ahead, foundation models trained on diverse immunotherapies and refined with causal−inference and uncertainty−quantification methods may support more mechanistically interpretable potency predictions and manufacturing guardrails (264), but these approaches remain developmental and will require robust risk−based regulatory frameworks and data−governance structures before routine use. Emphasis on risk-based regulatory frameworks and the development of interpretable AI systems will continue to shape the regulatory landscape for AI applications in immunotherapy potency assessment (236). Overall, progress toward broader acceptance of AI/ML in immunotherapy potency assessment will depend on demonstrable model interpretability, transparent documentation of training data and performance across diverse populations, robust change−control strategies for model updates, and governance frameworks that safeguard patient privacy and data integrity in multi−site collaborations.

## Regulatory agency frameworks for potency assay technologies

Recent regulatory guidance documents have emphasized need for more physiologically relevant potency assays with minimal artificial perturbations yet comprehensive characterization. The EMA guideline on potency testing states that “appropriately designed potency assays provide an accurate, reliable and consistent demonstration of the biological activity of the active ingredient” and emphasizes that potency assays should be “based on a defined biological effect as close as possible to the mechanism(s) of action/clinical response” (EMA, 2016). Similarly, the FDA’s 2023 draft guidance on potency assurance for cellular and gene therapy products emphasizes that “potency assays and their corresponding acceptance criteria should be designed to make meaningful contributions to potency assurance by reducing risks to product potency”. FDA describes potency assurance as multifaceted risk reduction (265).

Assays should reflect intended biological activity, but direct potency-efficacy correlation is not required. Rather, if a product is demonstrably efficacious and of acceptable risk-benefit, then a potency assay that quantitatively measures relevant biological function can be appropriate, even without direct correlation to clinical outcome. What needs demonstration is the specific capacity of the product to achieve a given result (266). Nonetheless, new analytical possibilities and methods of AI enhanced data correlation are increasing interest in correlative studies linking *in vitro* measurements to patient outcomes. The FDA’s Real-World Evidence Program has been developed to support use of real-world data (RWD) and real-world evidence (RWE) in regulatory decision-making, with the 21st Century Cures Act placing additional focus on these types of data to support regulatory decisions (248). The FDA’s computer software assurance guidance emphasizes importance of risk-based approaches to software validation that can accommodate the unique characteristics of AI systems while maintaining compliance with current Good Manufacturing Practice (cGMP) requirements (267).

The European Medicines Agency (EMA) has similarly advanced regulatory thinking through its Committee for Advanced Therapies (CAT) guidelines, that specifically addresses complexities of cellular immunotherapy potency assessment. The EMA 2024 safety reviews of CAR T-cell therapies included examination of secondary malignancies related to T-cells for six approved CAR T-cell medicines (268). Recent International Council for Harmonization (ICH) developments have focused on harmonizing requirements across global regulatory jurisdictions. The ICH Q5A(R2) guideline on viral safety evaluation for biotechnology products derived from cell lines was finalized in 2024, with the revised guidance now bringing into scope cellular and gene therapy products (269).

The increasing sophistication of immunotherapy potency assays presents novel regulatory challenges that agencies are actively addressing. The integration of multi-modal data sources and AI-powered analytics raises questions about validation requirements, algorithm transparency, and ongoing performance monitoring that extend beyond customary analytical validation paradigms (270). Explainable AI has become a critical requirement for regulatory acceptance. The FDA has emphasized the importance of algorithm interpretability and the ability to provide clear rationales for potency determinations (271).

Implementing a “digital twins” strategy for immunotherapy manufacturing has gained regulatory attention as a potential framework for integrating real-time potency monitoring with process control systems (246). The FDA has discussed digital twins as “in silico” representations or replicas of an individual that can dynamically reflect molecular and physiological status over time” in the context of drug development (272). Personalized medicine considerations are driving regulatory thinking toward more flexible potency frameworks that can accommodate patient-specific therapeutic optimization. The path forward will likely involve collaborative development between industry, regulatory agencies and academic institutions to establish best practices and validation standards. ICH work on harmonizing technical requirements across jurisdictions, combined with individual agency initiatives, creates foundations for consistent international approaches to AI validation (273). Future regulatory frameworks are expected to incorporate adaptive approaches that can evolve with technological advancement while maintaining appropriate oversight (270).

## Analytical validation principles for cell-based potency assays

Cell−based potency assays for solid−tumor immunotherapies are typically validated in line with ICH Q2(R2) and ICH Q6B expectations for biological assays, with adaptation to accommodate challenges such as biological variability, absence of an absolute “true potency” standard and product instability. Validation focuses on establishing fitness-for-purpose through systematic evaluation of accuracy, precision (repeatability and intermediate precision), specificity, linearity and range, robustness and ongoing system suitability. Key performance characteristics for accuracy include closeness of measured to nominal relative potency. Intermediate precision is tested across runs, operators and instruments while robustness is tested according to predefined variations in critical method parameters. For functional assays relevant to solid tumors (e.g. cytotoxicity, cytokine release, or 3D co−culture readouts), these characteristics have usually been established using a qualified reference standard, parallel−line or non−linear regression models. Predefined criteria for model fit and parallelism, and prospective estimation of assay variability support setting meaningful system−suitability and lot−release limits (274).

Inter−laboratory transferability is particularly important for multicenter solid−tumor programs, where consistent potency assessment underpins comparability across manufacturing sites and clinical studies. Transfer is commonly supported by cross−site bridging studies that evaluate bias and precision between laboratories using shared reference standards. Harmonized protocols and identical statistical models help establish predefined acceptance criteria for agreement. Together, these analytical validation principles applied with increasing stringency from early phase method suitability to late-phase full validation ensure that cell−based potency assays used in solid−tumor immunotherapy provide reproducible, mechanism−relevant measurements that are suitable for GMP lot release, stability studies and product comparability assessments (70, 266).

## Economic sustainability

The development and implementation of immunotherapy potency assays substantially impact the commercial viability of cellular therapies. Labor-intensive laboratory procedures for manual execution of Critical Quality Attribute (CQA) testing bring a significant cost burden introducing scheduling bottlenecks that increase manufacturing expenses and delay time-to-market. Over 200 labor hours were required per batch/lot with labor accounting for 71% of the total batch production costs (275).

Economic evaluations indicate significant variation in production costs reflecting the manufacturing model employed. Per-product manufacturing costs range from approximately $60,000 to $100,000 in academic settings, with quality testing representing a substantial variable component. In commercial settings, GMP-compliant release and potency packages commonly reached five-figure totals per batch depending on assay complexity and turnaround times (276, 277). For comprehensive treatment episodes requiring testing, monitoring and supportive care, total costs can exceed $1 million per patient (278). High-content imaging systems, multi-parameter flow cytometers, and specialized culture facilities can require initial capital investments exceeding $2–5 million, with ongoing maintenance and calibration costs adding 10-15% annually (279). Regulatory compliance costs compound these direct analytical expenses, with validation studies, documentation requirements and ongoing QC activities adding substantial overhead. Current regulatory frameworks require comprehensive potency and viability testing to ensure that each batch of products meets the highest quality standards, with potency assays being fundamental for comparability studies, process validation, and stability testing (280). A recent analysis of FDA-approved cell therapy products revealed that an estimated 104 total potency tests have been used across 31 approved products, with individual products averaging 3.4 potency tests each, indicating extensive validation requirements (281).

The integration of advanced technologies offers significant opportunities for reducing potency assessment costs while improving analytical capabilities and throughput. Automation technologies, including robotic liquid handling systems and automated imaging platforms, can reduce labor requirements by 60-80% while improving reproducibility and reducing human error. There are several process parameters to be influenced by next-generation strategies (3). Artificial intelligence and machine learning integration introduce opportunities for cost reduction. AI-powered image analysis can provide more comprehensive and objective results while reducing analysis time from days to hours. Machine learning algorithms capable of integrating multimodal data can facilitate discovery of new meta-biomarkers (202) with potential for achieving equivalent or superior analytical performance using 70-80% fewer experimental datapoints (282). Real-time monitoring, without labels or invasive techniques, e.g. impedance-based assays, can readily halve the cost of labeled assays while adding continuous monitoring capabilities (187). Sophisticated analytical capability investment isn’t merely a technical upgrade but a strategic imperative for sustainable competitiveness in the evolving immunotherapy landscape (283). More complex yet responsive lean manufacturing platforms (284) and quality by design approaches (285) ultimately provide returns on investment by establishing next-generation immunotherapy potency assays. Recent regulatory developments have created supportive frameworks for implementing advanced potency assessment technologies, with agencies recognizing limitations of traditional approaches and actively encouraging innovation through updated guidance documents and collaborative initiatives (265). Products supported by advanced analytical capabilities, may create additional economic value through accelerated approval pathways and market exclusivity extensions (286). While the preceding sections have focused on *in vitro* and ex vivo frameworks, the critical challenge lies in ensuring these measurements meaningfully reflect the product’s biological potential *in vivo*. For CAR-T therapies, this requires assessing functional attributes beyond simple cytotoxicity, since trafficking, persistence, and microenvironment resilience co-define the cell’s capacity to execute its therapeutic program within the patient.

## *In vivo* CAR T-cell engineering

*In vivo* CAR T-cell generation represents fundamental reconceptualization, transforming cellular immunotherapy into a directly administered gene therapy (287, 288). This categorical transition requires comprehensive redefinition of potency assessment frameworks that diverge substantially from established ex vivo CAR T-cell production, invoking distinct regulatory expectations aligned with gene therapy guidance rather than cellular therapy frameworks.

### Vector-centric potency assessment

Viral vectors (lentivirus, adeno-associated virus) or non-viral delivery systems (lipid nanoparticles, polymeric carriers) encoding CAR transgenes are administered systemically, targeting and transducing the patient’s endogenous T-cell compartment *in situ* (287, 288).

Under current FDA cellular and gene therapy guidance, *in vivo* CAR T approaches that use directly administered vectors are regulated as gene therapies because the vector formulation, rather than harvested cells, constitutes the drug product (28, 289). This regulatory reclassification triggers application of dedicated FDA and EMA gene therapy guidance, notably the ICH S12 “Nonclinical Biodistribution Considerations for Gene Therapy Products” (290) and the 2024 “Considerations for the Development of Chimeric Antigen Receptor (CAR) T Cell Products” guidance (28), that emphasize vector characterization, biodistribution, genotoxicity assessment, and real-time pharmacodynamic monitoring as key components for the development and benefit-risk evaluation of these products. Pre-infusion product characterization is eliminated (8), since the vector system itself constitutes the regulated product rather than manufactured cells (288). Traditional ex vivo-manufactured CAR T-cell therapies have been assessed using *in vitro* assays, such as transduction efficiency, cytotoxicity and cytokine secretion, but these metrics offer only limited insight for the performance of CAR T-cell generated directly *in vivo*.

### Pre-administration quality control

For *in vivo* CAR T generation, early stages of potency-related assessments center on vector quality, including the functional capacity of the delivery system to achieve efficient, specific, and safe T-cell transduction upon administration (28, 250, 287).

### Functional titer and transduction efficiency

Vector titer alone provides insufficient potency information; functional titer, measured as transducing units per milliliter on primary T-cell targets, represents the core metric. Standardized assays employ healthy donor-derived primary human T cells as surrogates for patient T cells, exposing these to defined vector doses and quantifying CAR expression via flow cytometry 48–72 hours post-exposure (75). For lentiviral (LV) systems, transduction efficiency benchmarks of ≥30-50% CAR+ cells at multiplicity of infection (MOI) 5–10 represent typical acceptance criteria (75, 291). For Adeno-Associated Virus (AAV) vectors, lower efficiencies (10-20%) reflect episomal nature but require reproducibility across manufacturing lots to ensure consistent clinical performance (110, 292).

### Targeting specificity and off-target risk

Unlike ex vivo manufacturing where off-target transduction is inconsequential, *in vivo* delivery risks transducing hepatocytes, endothelial cells, hematopoietic stem cells, or other non-target populations, creating safety liabilities, e.g. ectopic CAR expression and insertional mutagenesis in long-lived progenitor cells (287). For T-cell-targeted vectors, CD3-, CD4-, CD5-, or CD8-directed lentiviral pseudotypes or antibody-conjugated lipid nanoparticles, potency assays must demonstrate T-cell selectivity (288). Multi-lineage cell panels including hepatocytes, B cells, monocytes, and CD34+ hematopoietic progenitors undergoing parallel vector exposure need to achieve flow cytometric or qPCR detected selectivity ratios (target:off-target) exceeding 100:1 for clinical translation (293–295).

### Genetic integrity and *in vitro* surrogate functional readouts

Next-generation sequencing of vector genomes confirms CAR sequence fidelity, absence of mutations, proper regulatory element function, and lack of recombination products or replication-competent viral particles (292). Following vector-mediated transduction of primary T cells, functional assays assess resultant CAR+ T-cell activity as surrogate for *in vivo* performance, including target-specific cytotoxicity against patient-derived tumor organoids (227), cytokine secretion via multiplex assays measuring IFN-γ, TNF-α, IL-2, and granzyme B, proliferation capacity, and memory phenotype retention (8, 227, 293). Critically, these *in vitro* surrogates may predict but do not necessarily guarantee *in vivo* performance, as patient-specific factors including T-cell fitness, tumor burden, and immunosuppressive microenvironments profoundly influence outcomes.

### *In vivo* pharmacodynamic monitoring: real-time potency assessment

True product potency becomes expressed only post-administration within the patient and this requires validated quantifiable *in vivo* biomarkers enabling real-time monitoring across defined timepoints (28). Digital droplet PCR (ddPCR) provides ultrasensitive detection of CAR transgene copies in peripheral blood, enabling quantification with as few as 0.01% of total T cells (8). Serial sampling at days 3, 7, 14, 21, and 28 post-administration captured CAR T-cell generation kinetics, expansion dynamics, and persistence (75). Integration site analysis via ligation-mediated PCR can assesses clonal diversity and identify any potential insertional oncogenesis events requiring long-term safety monitoring per FDA guidance (28, 296). Beyond serial blood sampling approaches, non-invasive imaging technologies enable continuous monitoring without patient sampling. Positron emission tomography using radiolabelled (^89^Zr) CD19 ectodomain probes visualized CAR-T biodistribution within 24 hours, detecting accumulation in spleen, bone marrow, and tumor sites with high signal-to-noise ratios (297). Complementary modalities include nanobubble ultrasound and magnetic resonance imaging for magnetically guided delivery, providing dynamic persistence data without invasive procedures (298, 299). These label-free approaches transformed potency assessment from discrete timepoint sampling to continuous *in vivo* functional evaluation (300).

### Flow cytometric CAR expression and functional biomarkers

High-dimensional flow cytometry and mass cytometry (CyTOF) panels (≥20–40 parameters) simultaneously quantify CAR surface expression levels, T-cell subset distribution (CD4+ vs. CD8+), memory differentiation states, activation markers, exhaustion markers (PD-1, TIM-3, LAG-3, TOX, CD39), and proliferation indicators (301). For mRNA-LNP platforms delivering transient CAR expression, CAR levels typically peak at 24–72 hours post-administration returning to baseline by day 5-7, calling for sampling strategies adapted specifically to the delivery modality (302). Surrogate markers of CAR T-cell function may provide efficacy-linked potency readouts. In oncology applications, circulating tumor DNA (ctDNA), tumor-specific markers, or imaging-based response assessment e.g. iRECIST (303) can provide therapeutic activity surrogates. Multiplex serum cytokine analysis can capture systemic CAR T-cell activation; in particular, IFN-γ, TNF-α, and IL-2 elevation indicating functional CAR engagement, while IL-6, IL-1β, and IL-10 increases signal cytokine release syndrome risk requiring clinical intervention (8, 207, 293, 304).

### Biodistribution and tumor infiltration assessment

For solid tumor applications, comprehensive biodistribution studies in preclinical models (followed by clinical monitoring via tumor biopsies) assess CAR T-cell infiltration, spatial distribution within tumor microarchitecture, and functional status relative to tumor cells (305, 306). Imaging mass cytometry (IMC) with 40+ simultaneous markers and multiplex immunohistochemistry can quantify CAR T-cell positioning. Spatial potency metrics uniquely relevant to solid malignancies can be cells within 20 μm of tumor targets maintaining effector phenotypes (granzyme B+, Ki67+, IFN-γ+) while those >50 μm exhibiting exhaustion markers (PD-1+, TIM-3+, reduced granzyme B) (240, 305). CAR T-cell infiltration depth into 3D tumor spheroid cores directly correlated with cytotoxic efficacy, with spheroids >400 μm diameter exhibiting hypoxic cores resistant to CAR T-cell penetration and killing (240).

### Lipid nanoparticle potency assay considerations

Potency assessment for LNP systems focuses on comprehensive characterization of both the nanoparticle formulation and the resulting cellular response. Key quality control parameters include precise particle size distribution maintained between 70–90 nm, a polydispersity index below 0.2 nm indicating uniform particle size, high encapsulation efficiency exceeding 95%, and confirmed mRNA integrity through bioanalyzer analysis. Beyond these physical characteristics, testing verifies targeting ligand functionality through ELISA assays that confirm both successful antibody conjugation to the nanoparticle surface and retained antigen-binding capacity for cell-specific delivery. Functional assessments have employed pH-sensitive fluorescent probes to quantify endosomal escape efficiency, ensuring that the mRNA cargo successfully reaches the cell’s cytoplasm where translation can occur. Additionally, time-course studies documented the transient expression kinetics characteristic of mRNA-based approaches, with CAR protein expression typically peaking at 24–72 hours post-delivery and declining to baseline by days 5-7. This temporary expression pattern can be particularly advantageous, as it allows for repeat dosing strategies while potentially minimizing the long-term safety concerns associated with permanent genetic modifications (293, 302). Bimbo et al. (2025) showed that CD7-targeting combined with anti-CD3 activation ligands on LNPs enabled DNA and mRNA co-delivery, achieving stable CAR expression without requiring prior T-cell activation, making quiescent T cell targeting feasible (294). The modular design of LNP platforms enables ongoing improvements in CAR technology development (307). Dual mRNA strategies co-delivering more potent CAR with checkpoint inhibitors (anti-PD-1 scFv) or cytokines (IL-15) will require expanded potency assessment frameworks for solid tumor applications, as they simultaneously address multiple immunosuppressive mechanisms (122).

### Viral vector and polymeric nanocarrier systems

For AAV and targeted LV vectors, potency frameworks have integrated capsid/envelope protein functionality (cell-binding assays confirming receptor engagement), genome packaging efficiency (qPCR quantifying full vs. empty capsids, with >80% full particles required for clinical acceptance). Integration site risk analysis validates the integration site avoids oncogenes, promoter regions, and tumor suppressor loci. Immunogenicity assessment measures pre-existing neutralizing antibodies and post-administration anti-vector immune responses that could limit repeat dosing (110, 295, 296). Polymeric nanocarriers offer biodegradable alternatives with tuneable release kinetics, requiring distinct characterization including polymer degradation rates, transgene stability under physiological conditions, and sustained expression profiles (308).

### Strategic imperatives

For successful regulatory approval under evolving FDA gene therapy guidance (28), *in vivo* CAR T developers must demonstrate: (i) quantitative correlations between vector-mediated transduction efficiency in surrogate assays and clinical CAR T-cell generation, expansion, and function; (ii) minimum CAR+ T-cell thresholds at specified timepoints predictive of therapeutic efficacy; (iii) off-target transduction limits, insertional mutagenesis monitoring, and biodistribution constraints within potency frameworks per S12 guidance requirements (290); and (iv) platform-specific validation recognizing that LNP, AAV, and LV systems require distinct potency strategies reflecting unique delivery mechanisms, expression kinetics, and safety profiles (288). The field stands at a pivotal juncture where robust, validated potency assays will determine whether *in vivo* CAR T generation fulfills its promise of democratizing cellular immunotherapy for solid tumor patients.

## Plying dual pathways: ex vivo and *in vivo* potency assays

While *in vivo* CAR T-cell generation represents an exciting technological frontier bringing advantages in manufacturing speed and cost reduction, the primacy of ex vivo manufacturing approaches in current potency assessment frameworks reflects several critical scientific, clinical, and regulatory realities. A Proven Clinical Foundation has emerged from seven FDA-approved CAR T-cell products “all manufactured ex vivo” that have demonstrated remarkable clinical efficacy in relapsed/refractory hematologic malignancies; complete response rates often ranging from 39-98% with durable remissions extending beyond five years. This established clinical success has provided validated frameworks for potency assessment, manufacturing quality control, and regulatory compliance that have been refined through thousands of patient treatments (309). In contrast, *in vivo* CAR T-cell generation remains investigational, with zero approved products and limited human clinical data, making potency assessment frameworks inherently speculative rather than evidence-based. Critically, the ex vivo approach provides comprehensive pre-infusion quality control (310) and batches failing acceptance criteria are rejected before reaching patients, eliminating exposure to potentially ineffective or unsafe products. *In vivo* approaches shift quality control from pre-administration product testing to vector characterization and post-administration monitoring strategies (287, 288). While *in vivo* CAR+ T-cell generation cannot be verified before dosing, engineered viral vectors and nanocarriers undergo rigorous pre-clinical validation including cell-specificity testing through targeted pseudotypes or ligand-directed delivery systems, off-target transduction assessment in multi-lineage cell panels, and integration site analysis via next-generation sequencing to characterize insertional mutagenesis risk profiles (287, 292). Molecular engineering innovations, including self-inactivating lentiviral vectors, site-specific integration using engineered nucleases, transient mRNA-based expression eliminating genomic integration entirely, and tissue-restricted promoters limiting ectopic CAR expression, may substantially mitigate safety concerns historically associated with gene therapy (287, 293). Real-time pharmacodynamic monitoring via ddPCR, flow cytometry, and integration site sequencing enables rapid detection of off-target transduction or aberrant clonal expansion requiring clinical intervention (8, 287). For *in vivo* CAR T-cell approaches the regulatory challenge lies not in the absence of quality control per se, but rather in establishing validated surrogate assays and post-administration monitoring frameworks that provide equivalent confidence in product safety and efficacy compared to traditional pre-infusion testing paradigms; a gap that ongoing clinical trials and evolving regulatory guidance aim to address (288).

### Ex vivo and *in vivo* engineered CAR T-cell complementarity

*In vivo* CAR T-cell generation depends critically on the patient’s endogenous T-cell population as the substrate for modification. Heavily pre-treated patients may present with absolute lymphocyte counts <500 cells/μL, a threshold below which therapeutic cell expansion may be insufficient (311), or exhausted T-cell phenotypes, or immunosuppression from active infections or recent chemotherapy (287). Since *in vivo* transduction efficiency correlates directly with baseline T-cell counts and functional capacity (75, 312) such patients most in need, may least benefit from the *in vivo* engineering approach. This is particularly problematic in solid tumor settings, where an aggressive multi-line chemotherapy regimen preceding CAR T therapy routinely depletes and functionally compromises the T-cell compartment that *in vivo* systems require for success (313, 314). Additional concerns regarding off-target transduction of non-T cells, uncontrolled CAR expression, and potential immunogenicity of delivery vehicles will require systematic investigation in ongoing clinical trials.

Ex vivo manufacturing overcomes many of these patient-specific constraints through multiple mechanisms that are not feasible with *in vivo* approaches (128, 310, 315). The controlled ex vivo environment enables selective isolation and expansion of viable T-cell subsets even from severely lymphopenic patients (111). Optimized culture conditions can potentially rescue functionality from partially exhausted cells by incorporating exhaustion-reversing agents such as calcium signaling inhibitors (BTP-2, FK-506) or metabolic modulators (N-acetylcysteine). Under controlled ex vivo conditions, transient rest periods can reverse exhaustion phenotypes through epigenetic reprogramming (316–319). Increasingly sophisticated CAR T-cell engineering approaches can currently exceed *in vivo* delivery capabilities. Effective solid tumor CAR T cells often require simultaneous modifications including CAR expression, PD-1 or TGF-ß receptor knockout, chemokine receptor insertion (CXCR1/2 for trafficking), cytokine armoring (IL-15, IL-18), and suicide gene incorporation for safety (94). The genetic payload requirements for such multi-gene engineering (often >15 kb) exceed the packaging capacity of AAV vectors (<4.7 kb) (320) and challenge the efficiency of current LNP-mediated delivery systems, which achieve <1% stable integration without nucleases. Ex vivo platforms routinely accomplish these complex modifications through sequential transductions, electroporation, or CRISPR-mediated editing with comprehensive validation at each step (94).

There is likely to be a future where ex vivo and *in vivo* approaches will occupy complementary niches defined by clinical context and therapeutic objectives. Ex vivo manufacturing will likely remain preferred for scenarios requiring complex genetic engineering (multi-transgene constructs, safety switches), allogeneic products, precise dosing in vulnerable populations, and applications where upfront manufacturing time is acceptable (128, 321). *In vivo* generation may prove advantageous for rapidly progressive cancers requiring immediate intervention, resource-limited settings, chronic diseases requiring repeat dosing, and indications where controlled transient expression is desirable (287, 322). The field’s trajectory will ultimately be determined by forthcoming clinical trial data demonstrating comparative efficacy, safety, and practical feasibility. Early-phase human trials evaluating *in vivo* CAR T generation for hematologic and autoimmune indications are underway (323), though clinical efficacy in solid tumor applications, where barriers to CAR T success are most pronounced, remains undemonstrated (287). A rational, evidence-based therapeutic landscape will likely integrate both modalities, selecting the optimal approach based on individual patient characteristics, disease biology, and healthcare infrastructure.

## Strategic analytical integration for potency assay deployment

The convergence of multiple technological advances creates unprecedented opportunities to develop integrated analytical platforms capable of addressing the full spectrum of immunotherapy potency assessment requirements. Future research should prioritize integrating spatial biology insights with real-time monitoring capabilities, to enable continuous assessment of therapeutic cell behavior within physiologically relevant 3D environments. Recent advances in spatial profiling technologies have provided unprecedented analysis of complex cellular interactions uncovering novel spatial signatures, including univariate distribution patterns, bivariate spatial relationships, and higher-order structures that substantially enhance our capacity to evaluate physiologically relevant treatment strategies in solid tumor microenvironments (20, 153).

Artificial intelligence integration presents transformative opportunities for developing predictive models that can extrapolate from early *in vitro* observations to long-term clinical outcomes. Advanced machine learning architectures, particularly those incorporating deep learning approaches, can leverage global datasets while preserving data privacy, enabling collaborative development of robust predictive algorithms across international research networks (316). The development of AI-driven biomarker discovery frameworks using contrastive learning has considerable potential for identifying predictive biomarkers that can guide treatment selection. Pattern recognition across previously intractable diverse datasets, facilitates discovery of subtle yet clinically meaningful relationships between cellular phenotypes and therapeutic outcomes.

Technology development priorities must emphasize standardization and harmonization to facilitate widespread adoption while maintaining analytical rigor. The development of reference standards, calibration materials, and inter-laboratory proficiency programs will be essential for ensuring reproducibility and enabling regulatory acceptance of advanced analytical approaches (317). Collaborative platforms that share analytical protocols, validation datasets, and best practices will accelerate technology maturation while reducing individual development costs. Establishing consensus methodologies across research institutions and manufacturing facilities represents a critical prerequisite for transitioning sophisticated analytical technologies from research applications to clinical manufacturing environments.

Emerging technologies including quantum computing applications, advanced sensor integration, and next-generation sequencing platforms stand to further enhance potency assessment capabilities (318). Integrating synthetic biology, nanotechnology, and advanced materials science will innovate potency assessment approaches to overcome limitations in analytical sensitivity, throughput and biological relevance to address previously intractable analytical challenges (324).

## Regulatory landscape and clinical evidence generation

The FDA’s 2024 guidance agenda specifically focused on cellular and gene therapy products, with new guidance documents addressing considerations for chimeric antigen receptor T cell products and human gene therapy products incorporating human genome editing (28). Early engagement with agencies is advised to establish clear validation pathways for novel analytical approaches and well-designed correlative studies to demonstrate clinical relevance. This proactive regulatory engagement improves alignment between analytical development programs and evolving regulatory expectations, reducing the risk of expensive late-stage modifications to established analytical methods.

Complementing the FDA’s 2024 guidance, the EMA’s comprehensive guideline on investigational ATMPs came into effect in July 2025, establishing stage-appropriate expectations for potency assessment throughout clinical development (325). Notably, there is adoption of a more flexible approach compared to earlier drafts, as it now specifies that “a suitable potency assay should be in place when material for the first-in-human clinical trial is produced,” removing a previous requirement for full validation prior to confirmatory trials (317). This revision acknowledges the inherent complexity of cellular product mechanisms of action and the limited manufacturing experience available at exploratory trial stages. The EMA guideline emphasizes a risk-based approach to analytical method development, recognizing that while safety-related assays must be fully validated before first-in-human studies, potency assays can evolve iteratively as manufacturing experience accumulates and clinical understanding deepens. Growing alignment between EMA and FDA regulatory philosophies creates an increasingly harmonized international framework where analytical rigor is balanced with practical feasibility (**Table 4**). Nonetheless, developers must still carefully navigate jurisdiction-specific nuances in validation expectations and documentation requirements.

**Table 4:** Regulatory guidance alignment for potency assay strategy.

Regulatory body	Guidance document	Key recommendations for potency	Timeline requirements	Validation standards	Implications for assay selection	References
ICH (International Council for Harmonisation)	Q5A(R2) Viral Safety Evaluation of Biotechnology Products Derived from Cell Lines (Finalized 2024, now includes CGT)	Updated to explicitly include cellular and gene therapy (CGT) products; Viral safety assessment: adventitious agents, endogenous retroviruses; Applies to both ex vivo cellular manufacturing and vector production	Viral safety studies required before Phase 2/3 scale-up	Harmonized testing across FDA, EMA, PMDA (Japan); PCR-based viral detection, infectivity assays	Manufacturing quality: Viral testing not a potency assay but mandatory release criterion; Residual vector testing for CAR products critical for safety profile	(269)
ICH (International Council for Harmonisation)	Q6B Specifications: Test Procedures and Acceptance Criteria for Biotechnological/Biological Products (Step 4, 1999; principles still applicable)	Specifications should include identity, purity, potency, quantity, and safety tests; Potency assays measure biological activity using appropriate analytical procedures; Acceptance criteria based on manufacturing consistency and clinical data	Specifications defined before Phase 3; Justified based on accumulated batch data	Principles apply to cellular therapies though originally written for recombinant proteins; Emphasis on consistency and trend analysis	General framework: Core release assays define acceptance criteria based on Phase 2 manufacturing experience; Supporting assays provide additional product characterization; Exploratory assays inform process understanding but not required for batch release	(326)
EMA (European Union)	Guideline on Potency Testing of Cell-Based Immunotherapy Medicinal Products (EMA/CAT/271032/2015, adopted 2016)	Potency assays must demonstrate biological activity as close as possible to mechanism of action and clinical response; Appropriately designed assays provide accurate, reliable, and consistent demonstration of active ingredient activity; Multiple assays acceptable to address multifactorial complexity of cellular therapies; Matrix approach: functional + phenotypic + mechanistic assays	Qualified assay by Phase 2; Validated assay for Marketing Authorization Application (MAA); Comparability required for manufacturing changes	ICH Q6B principles adapted for biologics; Emphasis on consistency across batches rather than absolute potency correlation with efficacy	Core: MOA-linked functional assay (e.g., cytotoxicity, degranulation, phagocytosis depending on platform); Supporting: Identity/purity markers (flow cytometry phenotyping); Exploratory: Advanced mechanistic characterization (spatial biology, metabolic profiling)	(29)
EMA (European Union)	CAT Guideline on Quality, Non-clinical and Clinical Aspects of Gene Therapy Medicinal Products (Updates through 2024-2025)	Gene therapy products (including *in vivo* CAR vectors) require biodistribution and persistence data; Vector characterization: identity, purity, potency (infectious titer, transduction efficiency); Integration site analysis for integrating vectors (lentiviral, retroviral); Safety monitoring: insertional mutagenesis risk, replication-competent virus (RCR/RCV)	Nonclinical biodistribution before first-in-human; Long-term follow-up (LTFU) for patients receiving integrating vectors: 15 years	ICH Q5A(R2) viral safety principles; Vector copy number analysis; Stability studies for vector products	*In vivo* gene therapy potency: Vector infectious titer pre-administration release specifications; *In vivo* transduction efficiency post-administration potency assessment; Different from ex vivo cellular therapy paradigm	(327)
EMA (European Union)	Safety Review of CAR T-Cell Therapies: Secondary Malignancies (PRAC Meeting, January 2024)	Post-marketing surveillance identified T-cell malignancies in patients receiving approved CAR T products; Ongoing investigation: insertional mutagenesis vs. underlying disease risk; Emphasis on vector integration site monitoring and long-term genomic stability assessments	Enhanced pharmacovigilance for approved CAR T products; Registry enrollment for long-term follow-up	Enhanced safety monitoring; Vector integration site analysis increasingly scrutinized	Supporting assays gaining importance: Vector copy number (acceptance criteria tightening); Integration site analysis (clonality assessment); Genomic stability markers; Not release assays but critical for long-term safety monitoring	(268)
FDA (United States)	Potency Assurance for Cellular and Gene Therapy Products (Draft Guidance, December 2023)	Potency assays should reflect biologically relevant functional activity linked to mechanism of action (MOA); Multiple complementary assays recommended to capture multifactorial aspects of complex products; Direct correlation with clinical efficacy not required; assays must demonstrate relevant biological function; Risk-based approach: potency assurance is multifaceted risk reduction, not single-assay dependence	Qualified potency assay by Phase 2; Validated assay before pivotal Phase 3 trial; Process changes require comparability studies with validated potency panel	Fit-for-purpose validation adapted from ICH Q2(R1) principles; Accuracy, precision, specificity, linearity, range assessed per assay context; Stability-indicating assays emphasized	Core release assay: Simple, reproducible, MOA-linked functional test (e.g., cytotoxicity, cytokine release); Supporting assays: Phenotypic markers (CAR expression, memory subset), persistence biomarkers; Exploratory advanced assays: Metabolic fitness, spatial profiling, AI-enabled prediction (not required for release)	(265)
FDA (United States)	Considerations for the Development of Chimeric Antigen Receptor (CAR) T Cell Products (Draft Guidance, March 2024)	CAR expression level is critical quality attribute requiring quantification; Functional potency assays must assess target-specific cytotoxicity; Serial killing capacity and exhaustion markers (PD-1, TIM-3, LAG-3) recommended; Memory phenotype distribution (central memory, stem-like memory) associated with improved persistence; Safety assessment: vector copy number, residual vector contamination	Standardized cytotoxicity assay essential by Phase 2; CAR expression assay mandatory throughout development	ICH Q2 principles adapted; Emphasis on inter-lot consistency for comparability; Transduction efficiency and CAR expression stability required	Core: Target-specific cytotoxicity assay with antigen+ and antigen- controls; Core: CAR expression quantification (flow cytometry); Supporting: Memory phenotype, exhaustion markers; Exploratory: Metabolic fitness, persistence prediction models	(28)
FDA (United States)	S12 Nonclinical Biodistribution Considerations for Gene Therapy Products (Finalized May 2023)	Applies to gene therapy products including *in vivo* CAR T (AAV, LNP-mRNA vectors); Biodistribution assessment mandatory for products delivered systemically; Off-target transduction quantification in non-target tissues required; Potency for *in vivo* products measured post-administration, not pre-infusion	Biodistribution studies required before Phase 1 for systemic gene therapies; Repeat dosing requires additional biodistribution data	Gene therapy validation standards; PCR-based vector quantification; Functional assessment of *in vivo*-generated cells	Paradigm shift for *in vivo* CAR T: Vector titer/purity release specification; *In vivo* transduction efficiency potency endpoint (not ex vivo cytotoxicity); Biodistribution tracking via PET/bioluminescence imaging	(290)
FDA (United States)	Considerations for the Use of Artificial Intelligence to Support Regulatory Decision-Making for Drug and Biological Products (Draft Guidance, 2024)	AI systems supporting regulatory decisions must demonstrate transparency and explainability; Algorithm validation requires performance assessment across diverse datasets; Explainable AI (XAI) emphasized: ability to provide clear rationale for predictions; Risk-based validation approach for software assurance	AI algorithm validation before use in pivotal trials; Ongoing performance monitoring required post-approval	Software validation aligned with Computer Software Assurance (FDA 2022 draft guidance); Risk-based approach: higher scrutiny for release testing vs. exploratory use	AI-enabled potency prediction feasible but requires: Training data transparency; Algorithm interpretability (saliency maps, feature importance); Prospective validation studies; Currently more suitable for exploratory advanced assays than core release testing	(236)

Comparative summary of major FDA, EMA, and ICH guidance documents (2016–2024) relevant to potency assessment for cellular and gene therapy products, highlighting requirements for assay design, validation, and lifecycle implementation. The table organizes recommendations within a three−tier framework (core release assays, supporting assays, and exploratory advanced assays), emphasizing how evolving guidance has shifted expectations toward mechanism−linked functional assays, expanded genomic and integration−site safety testing, and incorporation of AI−enabled analytics under stringent validation and transparency principles.

A developer’s clinical evidence generation should prioritize establishing robust relationships between advanced potency measurements and clinical effects through carefully designed correlative studies. While direct correlation between potency assays and patient efficacy remains challenging and is not always achievable, these studies should focus on demonstrating biologically meaningful relationships that support product consistency and quality control. In contrast to traditional fixed validation paradigms, integration of real-world evidence collection with advanced analytical platforms creates opportunities for continuous validation and iterative refinement of potency assessment approaches, enabling data-driven optimization based on accumulated clinical experience (328).

Regulatory sandbox programs and pilot initiatives provide controlled environments for validating innovative potency assessment approaches without full regulatory burden, accelerating the translation of advanced technologies into routine practice (329). These programs allow systematic evaluation of novel analytical methods while generating evidence for broader regulatory acceptance. By reducing barriers to analytical innovation, regulatory sandbox frameworks encourage development of sophisticated measurement technologies that might otherwise be considered too risky for immediate clinical implementation.

International harmonization efforts continue to expand, with regulatory agencies collaborating to develop common standards and mutual recognition frameworks that facilitate global implementation of advanced potency assessment technologies (330). Addressing these challenges requires coordinated international efforts. For example, the recently established COST Action CA24114 (BTCs4ATMP, 2025-2029) aims to create a pan-European network promoting standardization of potency assay procedures and harmonization of quality control testing for ATMPs, including CAR T-cell therapies, through protocol sharing and collaborative validation studies across Blood, Tissue, and Cell processing centers, an initiative that addresses the critical need for consensus in potency assessment methodologies. Such collaborative networks provide essential infrastructure for developing validated consensus approaches applicable across diverse regulatory jurisdictions.

### Progressive implementation of potency assessment

Implementing advanced potency assessment technologies benefits from a phase-appropriate strategy aligned with product development stages and regulatory expectations (30, 331). For early-phase (Phase I/II) programs, laboratories should prioritize establishing core functional assays (cytotoxicity, cytokine release) and basic phenotyping (CAR+ percentage, memory markers), focused on obtaining biological activity and consistency rather than comprehensive mechanistic characterization. This foundational tier provides essential release criteria while conserving resources during stages when manufacturing processes are still evolving.

As programs advance to Phase II/III, potency panels should expand to include mechanism-of-action relevant assays and more sophisticated phenotypic characterization, incorporating multi-parameter flow cytometry and functional persistence indicators (332). Typically, full method validation occurs during late Phase III and process performance qualification, with acceptance criteria tightened based on accumulated manufacturing data and clinical correlations. This progressive approach balances analytical rigor with practical feasibility, enabling continuous improvement while maintaining regulatory compliance throughout the product lifecycle.

### Plying the potency panel from minimal to comprehensive

When designing potency panels, laboratories are often required to balance analytical comprehensiveness with practical resource constraints. For solid tumor immunotherapy products, a minimum viable potency panel typically comprises (i) a primary mechanism-of-action assay (demonstrating target-specific cytotoxicity or cytokine release), (ii) CAR or TCR expression quantification, and (iii) basic viability/identity markers (28). In contrast, comprehensive assay panels build on this foundation with multi-dimensional functional characterization (serial killing, exhaustion markers, metabolic fitness), advanced phenotyping (memory subset distribution, transcriptional profiling), and predictive assays (patient-specific tumor models, spatial profiling). The choice and timing of such assay expansion and the extent of characterization is ideally guided by product complexity, clinical development stage, and the availability of correlative clinical outcome data.

### Emerging capabilities and manufacturing evolution

There is a strong drive to implement multi-modal platforms that can seamlessly combine spatial biology, real-time monitoring, and artificial intelligence to provide comprehensive characterization of therapeutic cell products. These next-generation systems will systematically apply multiple analytical tools while addressing the multi-layered challenges of solid tumor immunotherapy through sophisticated 3D models that incorporate physiological gradients and environmental stressors. While integrated multi-modal platforms represent substantial capital investment, their implementation becomes economically compelling as ATMP production transitions from early phase small-scale to industrial scale, where comprehensive analytical sophistication offsets the prohibitive costs of batch failures and regulatory delays. Ultimately, the successful deployment of next-generation integrated potency assessment platforms will be determined not by their technical sophistication alone, but by the strategic alignment of implementation timelines with market maturation and production volumes sufficient to achieve cost-effectiveness. This economic reality necessitates careful consideration of implementation pathways that balance analytical ambition with practical manufacturing constraints.

Personalized medicine applications call for tailoring potency assessment approaches to individual patient characteristics and therapeutic requirements, a need that is especially pronounced in solid tumor immunotherapy given the inherent heterogeneity of tumor microenvironments and the variability in patient responses. Future platforms will likely integrate patient-derived organoid models with autologous therapeutic cells, enabling personalized optimization of treatment protocols while providing individualized potency thresholds based on patient-specific factors. The development of portable analytical platforms that can deliver advanced potency assessment in distributed settings could significantly enhance global accessibility. Contextual considerations may nonetheless govern strategy. Personalized, organoid-augmented potency assessment is most valuable where tumor ecology drives response heterogeneity, a consideration highly relevant to many solid tumors. It can be cost-effective when it prevents expensive failures and accelerates effective treatment. Conversely, allogeneic standardization remains the most scalable route for speed and access.

Ultimately, developers may favor a hybrid approach: a harmonized, mechanism-of-action-anchored potency backbone for all products, with selectively deployed, automation-friendly patient-context modules where the expected clinical utility advantage justifies the operational overhead. This hybrid approach may help mitigate technology risk by maintaining validated standard pathways while selectively deploying advanced capabilities where clinical evidence supports their use. Near-term implementations will likely focus on high-impact, automation-ready modules, while comprehensive patient-context platforms will emerge as manufacturing scales and regulatory pathways mature. In this phased implementation strategy, full personalization of potency assessment is not assumed to be universally appropriate; rather, greater assay complexity is introduced when justified by anticipated clinical value.

Digital manufacturing intelligence is poised to emerge through digital twin technologies that enable virtual representations of manufacturing processes and analytical systems, allowing predictive optimization and scenario modeling to enhance both efficiency and quality. These approaches will integrate real-time data streams with mechanistic models and AI-driven analytics, enabling improved predictive quality control and more adaptive manufacturing strategies.

Autonomous analytical ecosystems powered by advanced AI will enable continuous monitoring and optimization of potency assessment protocols to reduce human intervention while improving consistency and reliability (333). A long-term aspiration is that these systems will incorporate real-time self-validation capabilities and continuous learning algorithms that can adapt to new therapeutic modalities and manufacturing processes while maintaining regulatory compliance through built-in audit trails and decision transparency. Such autonomous systems represent the culmination of advances in artificial intelligence, sensor technology, and quality management principles, enabling unprecedented levels of analytical consistency and responsiveness to process variations.

The development of cost-effective analytical solutions guided by global accessibility considerations offers significant potential for democratizing access to advanced potency assessment capabilities Cloud-based analytical platforms that can provide sophisticated AI-powered analysis capabilities without requiring extensive local infrastructure represent promising approaches for enabling worldwide implementation of advanced analytical technologies. Distributed analytical architectures utilizing cloud-based platforms offer potential for ensuring that sophisticated potency assessment capabilities are not restricted to well-resourced institutions in developed markets, but successful implementation will require addressing challenges related to connectivity infrastructure, data sovereignty regulations, and local technical capacity (334).

Sustainability and environmental considerations are likely to increasingly influence the development of next-generation potency assays, with emphasis on reducing resource consumption, minimizing waste generation, and improving energy efficiency. Green analytical chemistry principles have the potential to guide the development of environmentally responsible potency assessment technologies. As the cellular therapy field matures, environmental sustainability may transition from peripheral consideration to central design principle (335), helping to ensure that analytical advancement does not impose unsustainable environmental burdens.

Educational and workforce development initiatives will be critical for ensuring successful implementation of advanced potency assessment technologies, requiring comprehensive training programs and competency frameworks that can prepare future analytical scientists for the challenges ahead. The increasing sophistication of potency assessment methodologies will benefit from a corresponding evolution in training strategies to ensure that the analytical workforce possesses the interdisciplinary skills likely to be required to implement and maintain these complex systems.

The convergence of these technological, regulatory, and implementation advances has the scope to advance immunotherapy potency assessment toward becoming a mature analytical discipline capable of supporting the next generation of cellular therapies. Through systematic application of advanced analytical tools, the field may be better positioned to address current limitations and contribute to establishing improved standards for therapeutic cell characterization that can help inform the development of more effective and accessible immunotherapy treatments for patients with solid tumor malignancies.

## Strategic implementation of the plying potency framework

The design of advanced therapy medicinal products, and their progression from preclinical studies into Phase I and II trials, inherently demands a potency strategy that becomes more structured and selective over time. Early development can justifiably rely on broader, exploratory measurements to understand biological potential, but clinical advancement requires progressively tighter control, consistency, and regulatory defensibility. Regulatory experience indicates that potency is rarely demonstrated by a single, all−encompassing assay; instead, agencies generally expect a CQA−anchored core assay with release criteria firmly linked to the dominant mechanism of action, supported by MOA−consistent secondary assays that contextualize biological activity.

The plying framework operationalizes this expectation through a tiered structure layered above a discovery base. For clinical entry and routine batch release, Core Release Assays provide the primary, MOA−linked potency readouts that form the backbone for comparability and regulatory confidence across the product lifecycle. Around this core, Supporting Characterisation Assays capture identity, phenotypic composition, selected functional attributes, and essential viability and purity parameters, offering a transparent minimum viable potency panel for early−phase programs. Supporting assays complement but do not duplicate the core; selected assays may be promoted to core status when predefined evidence thresholds are met, for example when a parameter shows stable, MOA−consistent correlations with CQAs or other predefined potency indicators (e.g. validated functional or biomarker surrogates of biological activity) and can be validated to the same standard as existing core assays, as outlined in FDA’s draft potency assurance guidance and reiterated by the American Society of Gene & Cell Therapy (ASCGT) (336) and International Society for Cell & Gene Therapy (ISCT) (337).

Additional biological complexity is addressed through Exploratory Advanced Assays that typically originate in discovery and preclinical research but are layered onto the control strategy only when their added predictive value justifies the increased complexity, cost, and validation burden. These assays include 3D spheroids and organoids, hypoxia−modulated culture systems, spatial profiling, label−free real−time cytotoxicity platforms, and AI−enabled analytics that interrogate persistence, trafficking, metabolic fitness, resistance mechanisms, and microenvironmental stress. Their principal “win” is the ability to reveal failure modes that core tests cannot detect, such as poor infiltration into hypoxic tumor cores or early exhaustion under low effector−to−target ratios and to generate mechanistically grounded biomarkers that can later be distilled into leaner core or supporting assays. Economically, these platforms can be cost−saving at program level by preventing late−stage failures and avoiding scale−up of products unlikely to succeed *in vivo*, even though they are more expensive than individual core tests.

At the apex of the framework, Translational Integration Modules link assay data across tiers with clinical and real−world evidence. Multi−modal models, including AI−driven analytics and digital manufacturing twins, integrate measurements from Core Release, Supporting Characterisation, and Exploratory Advanced Assays together with clinical covariates (tumor burden, microenvironmental features, prior therapies) to identify which combinations of readouts best track efficacy, durability, and toxicity. These modules convert many heterogeneous assays into a smaller number of decision−ready indices that support dose selection, comparability after process changes, and post−marketing risk management. They are particularly critical for *in vivo* CAR products, where potency cannot be fully defined by pre−administration testing and must instead be inferred from integrated models combining vector attributes, early pharmacodynamic biomarkers (e.g. ddPCR copy number, CAR expansion by flow cytometry, cytokine signatures), and clinical outcomes.

Each tier aligns with, but is not synonymous with, clinical development phases. Discovery and preclinical work primarily occupy the discovery base and early Exploratory Advanced space; Phase I entry requires at least one MOA−linked Core Release Assay with appropriate Supporting Characterisation, while Exploratory Advanced Assays run in parallel as correlative tools. During Phase II and Phase III, evidence from exploratory platforms and early clinical correlations can justify promotion of selected assays into the core or supporting tiers and provide inputs for Translational Integration Modules that become increasingly important for late−phase decision−making and post−approval lifecycle management. Promotion between tiers is guided by explicit rules of evidence: assays are moved upward only when they demonstrate reproducible, MOA−consistent association with critical quality attributes or predefined potency indicators and can meet phase−appropriate validation expectations.

The effectiveness of this strategy ultimately depends on transparent communication with regulators. Developers should clearly describe the assay selection logic, specifying which assays constitute the Core Release panel, which are Supporting Characterisation Assays that provide additional evidence and may later be upgraded, which are Exploratory Advanced Assays used to discover and refine biomarkers, and how Translational Integration Modules will be used to synthesize data across tiers and clinical phases. With an intentionally layered, evidence-driven plying framework, CAR potency testing aligns increasing analytical complexity with clinical maturity while preserving regulatory clarity and scientific depth.

## Conclusions

Future research initiatives are likely to prioritize predictive models that better connect *in vitro* potency measurements with clinical outcomes, for more accurate estimation of therapeutic benefit and optimization of treatment protocols. Establishing comprehensive databases that link potency readouts with longitudinal clinical data would provide a foundation for evidence−based potency assessment and continuous refinement of analytical methods. In parallel, advances in international regulatory harmonization will be essential to ensure that these analytical capabilities can be deployed consistently across global markets while maintaining uniform quality standards.

A comprehensive, integrated ecosystem of advanced analytical technologies can support the full immunotherapy lifecycle, from early discovery through clinical translation, commercial manufacture and post−market surveillance. Such integration can improve quality control, process optimization and therapeutic efficacy, helping to expand global access to advanced immunotherapies. By systematically plying potency assessment capabilities across the product lifecycle (particularly to address the distinctive challenges of solid−tumor immunotherapy), it becomes possible to achieve sustained improvements in patient outcomes while upholding the quality standards essential for safety and regulatory compliance.
